# Offline hippocampal reactivation during dentate spikes supports flexible memory

**DOI:** 10.1016/j.neuron.2024.08.022

**Published:** 2024-09-24

**Authors:** Stephen B. McHugh, Vítor Lopes-dos-Santos, Manfredi Castelli, Giuseppe P. Gava, Sophie E. Thompson, Shu K.E. Tam, Katja Hartwich, Brook Perry, Robert Toth, Timothy Denison, Andrew Sharott, David Dupret

**Affiliations:** Medical Research Council Brain Network Dynamics Unit, Nuffield Department of Clinical Neurosciences, University of Oxford, Oxford, OX1 3TH, UK

## Abstract

Stabilizing new memories requires coordinated neuronal spiking activity during sleep. Hippocampal sharp-wave ripples (SWRs) in the Cornu Ammonis (CA) region, and dentate spikes (DSs) in the dentate gyrus (DG) are prime candidate network events for supporting this offline process. SWRs have been studied extensively but the contribution of DSs remains unclear. By combining triple-(DG-CA3-CA1) ensemble recordings and closed-loop optogenetics in mice we show that, like SWRs, DSs synchronize spiking across DG and CA principal cells to reactivate population-level patterns of neuronal coactivity expressed during prior waking experience. Notably, the population coactivity structure in DSs is more diverse and higher-dimensional than that seen during SWRs. Importantly, suppressing DG granule cell spiking selectively during DSs impairs subsequent flexible memory performance during multi-object recognition tasks, and associated hippocampal patterns of neuronal coactivity. We conclude that DSs constitute a second offline network event central to hippocampal population dynamics serving memory-guided behavior.

## Introduction

Memories are stabilized during periods of sleep and rest ^1–4^. Decades of work have provided important insights into the underlying brain network mechanisms and have identified offline hippocampal activity as essential for this process ^5,6^. Central to our current understanding are hippocampal sharp-wave ripples (SWRs) that feature an intermittent, high-frequency (100 – 250 Hz) network event detected in the local field potentials (LFPs) of the CA1 region ^7–10^. During SWRs, the firing activity of CA1 principal cells is transiently modulated ^11,12^ and reactivates the population-level firing patterns expressed in previous waking experience ^13^. These offline spiking correlates have behavioral significance: suppressing CA1 neurons during SWRs impairs memory recall for recently acquired information ^14–16^. Conversely, prolonging SWRs or reinforcing the coordination between SWRs and neocortical activity promotes memory consolidation and subsequent behavioral performance ^17,18^. Hippocampal SWRs therefore constitute an offline network event important for memory-guided behavior. However, during sleep/rest periods the hippocampus exhibits another prominent network event: dentate spikes (DS), which are seen in the LFPs of the dentate gyrus (DG). To date, DSs have received little attention compared to SWRs. Accordingly, here we characterize the neuronal spiking dynamics nested in DSs with respect to SWRs and evaluate whether DSs constitute a second network event central to offline reactivation of waking firing patterns and subsequent memory-guided behavior.

The DG gates sensory information to the hippocampus, notably decorrelating these inputs into dissimilar neural patterns ^19–21^. This function may be crucial for the hippocampus to integrate multiple items in memory and to flexibly distinguish between stimuli with overlapping features. Within the DG, DSs represent intermittent, large amplitude network events recorded in the LFPs of the DG granule cell layer and are associated with increased spiking activity in dentate cells ^22–24^. However, across the literature both increased and suppressed spiking activity of CA principal cells have been reported ^22,24–28^, although, notably, some of these studies were in anesthetized ^25,27^ or head-fixed animals ^24,28^. Thus, here we further performed a systematic comparative assessment of DG and CA principal cell spiking activity during DSs versus SWRs in non-anesthetized, freely behaving mice.

To investigate the influence of DSs on hippocampal population activity and memory, we combined triple-(DG-CA3-CA1) site extracellular multichannel recordings and closed-loop optogenetic interventions in mice during active exploratory behavior and offline sleep/rest. We observed that during offline DSs, principal cell spiking transiently increased across the DG and CA regions of the hippocampus, nesting offline population-level activity patterns that are distinct from those in SWRs. Further, we report that the cell-to-cell coactivity seen during prior waking experience is reactivated during DSs (as well as SWRs). DS-nested neuronal activity is relevant to whole-hippocampus population dynamics and memory-guided behavior: closed-loop suppression of DG granule cell spiking selectively during offline DSs, but not SWRs, impairs subsequent flexible memory performance in hippocampal-dependent, multi-object recognition tasks. We propose that DSs constitute a second hippocampus network event that plays a complementary role to that of SWRs by supporting the offline reactivation of diverse population patterns of neuronal coactivity in support of memory-guided behavior.

## Results

### Firing activity of hippocampal neurons synchronizes during DS events

**Figure 1:**
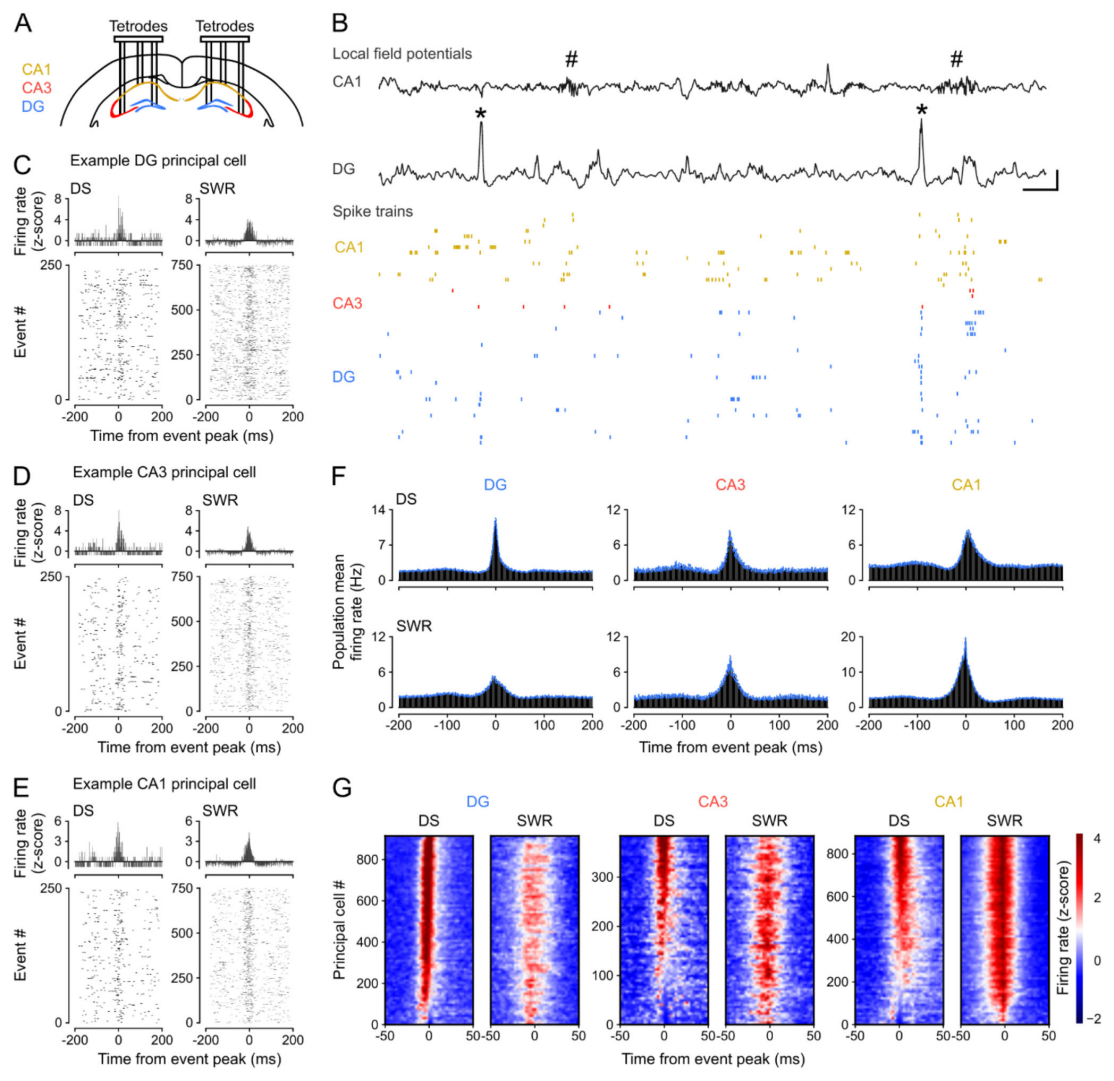
Dentate spikes recruit principal cell spiking across DG, CA3, and CA1. **(A)** Triple-(DG-CA3-CA1) ensemble tetrode recording allowed simultaneous monitoring of local field potentials (LFPs) and spiking activities. **(B)** Upper: raw wide-band CA1 and DG LFP traces (black) showing sharp-wave ripples (SWRs, hash symbols) in CA1 and dentate spikes (DSs, asterisks) in DG. Scale bars, 100 ms (horizontal), 1.5 mV for DG and 0.5 mV for CA1 (vertical). Lower: (color-coded) raster-plot of spike trains from CA1 (orange), CA3 (red), and DG (blue) principal cells (PCs, one cell per row). Shown is a few second sample of recording for clarity. **(C-E)** Spiking responses from single example DG (C), CA3 (D), and CA1 (E) principal cells. Upper: Z-scored peri-event time histogram (PETH) during DSs (left) and SWRs (right). Lower: corresponding raster plot showing event-related spiking responses (one event per row). **(F)** Group averaged firing rate PETHs for hippocampal PCs during DSs (top) and SWRs (bottom): DG (n=921), CA3 (n=388), CA1 (n=887) cells from 12 mice. Blue traces: mean ± SEM. **(G)** Heatmaps showing z-scored firing rates for the DG, CA3, and CA1 PCs shown in (F). For each heatmap: one cell per row, sorted (top-to-bottom) from the most activated (highest z-score at event peak, 0 ms, red) to the least activated (lowest z-score at event peak, blue) during DSs.

We first used triple-(DG-CA3-CA1) site tetrode recordings to monitor network events in the LFPs and the spike trains of neuronal ensembles from the dorsal hippocampus of mice during sleep/rest (Figures 1A,B and S1A-C; n = 12 mice). From these LFPs, we detected DSs in DG and SWRs in CA1 to compare the spiking activity of principal cells between these two types of network event. Across mice and recording sessions DS waveforms were highly consistent (Figure S1D,E). Both DG DSs and CA1 SWRs were of short duration (Figure S1F; median (IQR) duration: DSs = 42.4 (40.0 – 46.4) ms; SWRs = 47.5 (45.2 – 51.0) ms) and occurred intermittently (median (IQR) occurrence frequency: DSs = 0.40 (0.25 – 0.58) Hz; SWRs = 0.75 (0.26 – 1.26) Hz) during behavioral and LFP profiles indicative of sleep/rest (Figure S1G,H). These two network events rarely occurred simultaneously, consistent with previous reports ^22,29^, with the vast majority of DSs not expressed within ± 50 ms of a SWR (median (IQR): 92.5 (87.9 – 95.6) %; DS-SWR co-occurrence frequency: 0.03 (0.02 – 0.05) Hz; Figure S1I,J). We computed the firing responses of individual principal cells (n = 2,196 total recorded principal cells; CA1, 887; CA3, 388; DG, 921 cells; Figure S2A-F) with respect to the peak of either DSs or SWRs, excluding those temporal windows where both events co-occurred within ±50 ms. With the term “principal cells,” we refer to CA pyramidal cells and DG granule cells that constitute the dominant (hence “principal”) cell type in the hippocampus, exhibiting lower mean firing rates compared to local fast-spiking inhibitory cells (Figure S2E). In line with previous work, DG principal cells transiently increased their firing activity during DSs ^22,25^; and the activity of CA principal cells increased during SWRs (Figures 1C-G and S2G-P) ^8,30^. We further observed that DG principal cell firing increased during SWRs (Figures 1C,F,G and S2G-P); and that CA principal cells also increased their firing rate during DSs (Figures 1D-G and S2G-P), which contrasted with some earlier reports that CA principal cell firing is suppressed during DSs ^22,25^.

To quantify the magnitude of neuronal activation during DSs and SWRs, we calculated the proportion of DG, CA3, and CA1 principal cells that increased their firing rate beyond a given significance threshold, using the z-scored peri-event time histograms obtained for each of these two network events (Figure 1G). During DSs, the majority of DG (91%), CA3 (56%), and CA1 (61%) principal cells increased their firing rate more than three standard deviations above baseline (z-score > 3, p < 0.003; Figure S2G-I). During SWRs, a comparable proportion of principal cells significantly increased their firing activity beyond this threshold (Figure S2G-I). Hippocampal CA principal cells exhibited preferential activation during SWRs whereas DG principal cells exhibited preferential activation during DSs (Figure S2I). DG principal cell population typically fired before CA principal cell populations during DSs (Figure S2J-N). While DG and CA principal cells exhibited such a temporal relationship, both DSs and SWRs were associated with an overall transient increase in hippocampal spiking activity (Figure S2O,P).

**Figure 2:**
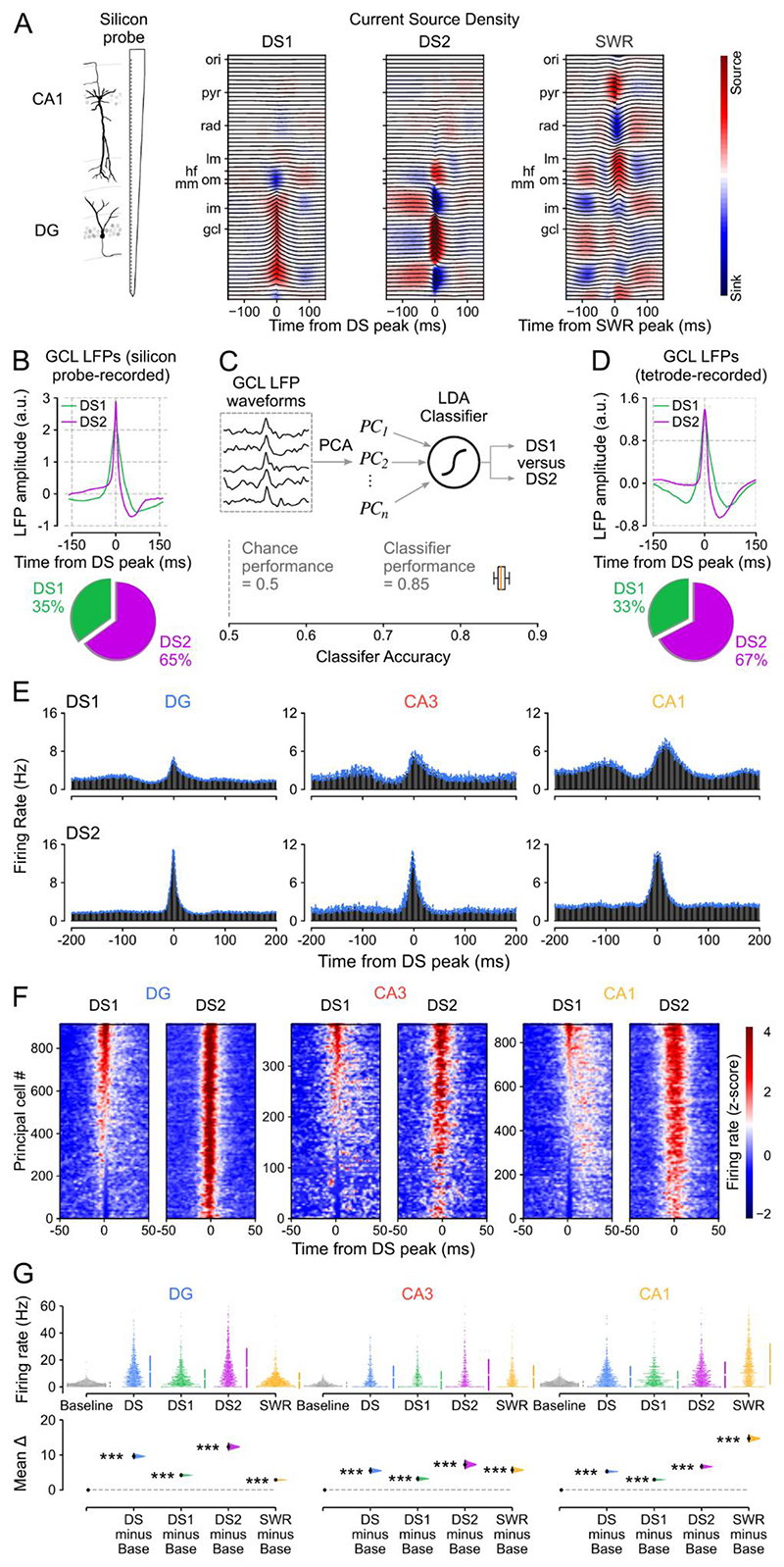
Hippocampal principal cell firing is higher in DS_2_ than DS_1_. **(A)** Left: Laminar (64-channel) silicon-probe recording allowed simultaneous monitoring of LFPs across hippocampal layers for current source density (CSD) analysis. Right: Example (radially organized) mean LFP traces (gray) with superimposed CSD profile (heatmaps) for type 1 (DS_1_) and type 2 (DS_2_) dentate spikes and SWRs (calculated from 2,231 DS events and 8693 SWR events in one mouse). Note the distinct CSD profiles reflecting the different transmembrane currents associated with DS_1_ versus DS_2_ versus SWR events. Hippocampal layers: oriens (*ori*); pyramidale (*pyr*); radiatum (*rad*); lacunosum-moleculare (*lm*); outer (*om*), middle (*mm*), and inner (*im*) moleculare; granulare (*gcl*). Hippocampal fissure (*hf*). **(B)** Upper: Shown for silicon-probe recorded DS_1_ and DS_2_ identified from their CSD profiles are example average granule cell layer LFP waveforms triggered by the peak of these events. Lower: in these recordings there was a higher proportion of DS_2_ than DS_1_ events (n=15,067 events, 3 mice). **(C)** Upper: we applied principal component analysis on the normalized granule cell layer LFP waveforms for all silicon-probe recorded DS events. We then used the principal components explaining 90% of the variance to train a linear discriminant classifier with the true labels (DS_1_ versus DS_2_) determined by the individual CSD profiles. Lower: the classifier performance (>85%) was significantly above chance level (50%) when tested on silicon-probe recorded LFP waveforms of unlabeled events. We used this classifier to next distinguish DS_1_ and DS_2_ from tetrode-recorded granule cell layer LFP waveforms (D). **(D)** Upper: Shown for tetrode-recorded DS events are the average granule cell layer LFP waveforms for DS_1_ and DS_2_ predicted label obtained from the silicon-probe-based classifier (C). Lower: these recordings also contained a higher proportion of DS_2_ than DS_1_ events (n=32,215 events, 12 mice). **(E)** Group averaged firing rate PETHs for tetrode-recorded DG, CA3, CA1 principal cells during DS_1_ and DS_2_ (as Figure 1F,G). Blue traces: mean ± SEM. **(F)** Heatmaps showing z-scored firing rates for the DG, CA3, and CA1 cells shown in (E). For each heatmap: one cell per row, sorted (top-to-bottom) from the most activated (highest z-score) to least activated (lowest z-score) during DS_1_ peaks. **(G)** Estimation plot showing the effect size for the differences in firing rate of DG, CA3, CA1 principal cells during all DS events, DS_1_ and DS_2_ events analyzed separately, and SWRs compared to equivalent (50 ms duration matched) baseline windows (Base) in which no DSs or SWRs occurred. Upper: raw data points (each point represents one cell), with the gapped lines on the right as mean (gap) ± s.d. (vertical ends) for each event. Lower: difference (Δ) in firing rate between Baseline windows and all DS, DS_1_, DS_2_, and SWR events computed from 5,000 bootstrapped resamples and with the difference-axis origin (dashed line) aligned to the baseline rate (black dot, mean; black ticks, 95% confidence interval; filled curve, sampling-error distribution). The test statistic is the mean difference, shown on the y-axis of the lower plot. P-values are from paired permutation tests, event versus baseline, ***P < 0.001. E-G show data from n=2196 hippocampal principal cells (DG: n=921, CA3: n=388, CA1: n=887) from 12 mice.

Previous studies identified two types of DS event (DS_1_ and DS_2_), based on the laminar profile of the transmembrane currents associated with the LFP expression of these network events ^22,24,26^. Therefore, we next asked whether principal cell firing responses differed between DS_1_ and DS_2_. However, localizing sinks and sources of currents across hippocampal layers requires applying Current Source Density (CSD) analysis ^31^ to the LFPs measured at evenly spaced sites from the CA1 oriens layer to the DG granule cell layer. Such a laminar profile is not accessible with tetrode recordings. To distinguish between DS_1_ and DS_2_ events, we therefore implanted linear silicon-probes spanning the somato-dendritic axis of CA1 principal cells and reaching the inferior blade of the DG in a separate group of mice (n = 3). Having performed silicon-probe recordings during sleep/rest, we applied CSD analysis to these LFPs measured over the radial extent of the hippocampus to identify DS_1_ versus DS_2_ according to their underlying profile of current sinks and sources (Figures 2A and S3A). These CSD-validated DS_1_ and DS_2_ events exhibited distinct DG granule cell layer LFP waveforms (Figures 2B and S3B). We then trained a linear discriminant analysis classifier to identify these CSD-validated DS_1_ versus DS_2_ events using only their DG granule cell layer LFP signal. When tested on the silicon-probe LFP dataset, the classifier achieved over 85% accuracy (Figure 2C). When next applied to the tetrode LFP dataset, the classifier-identified DS_1_ and DS_2_ events also exhibited distinct granule cell layer LFP waveforms (Figures 2D and S3C,D), which were consistent with those obtained in silicon-probe recordings (Figures 2B and S3B). In both (tetrode and silicon-probe) datasets, DS_2_ represented two-thirds of the DS events (median (IQR): 66 (61 – 73) %), thus constituting the dominant type. Leveraging this cross-dataset approach, we found that the firing response of DG and CA principal cells was stronger for DS_2_ than DS_1_ (Figures 2E-G and S3E-J). A greater proportion of CA principal cells showed firing activity below baseline during DS_1_ compared to DS_2_ (35% versus 11%; Figures 2F and S3K,L), providing insights into the previously documented DS-suppressed firing in some CA principal cells ^22,25,32^. Nevertheless, the average activity of principal cells in DS_1_ (and DS_2_) was significantly higher than their baseline firing (calculated outside of any DS and SWR events) during sleep/rest (Figures 2G and S3G,H) and their overall mean firing rate calculated over the whole recording day (Figure S3I,J). These results show that DS events (both DS_1_ and DS_2_) constitute transient network states that are qualitatively distinct from the sleep/rest epochs outside these events in terms of their capacity to increase spiking activity of individual principal cells distributed across hippocampal regions.

### DS events nest higher dimensional patterns of population coactivity

**Figure 3:**
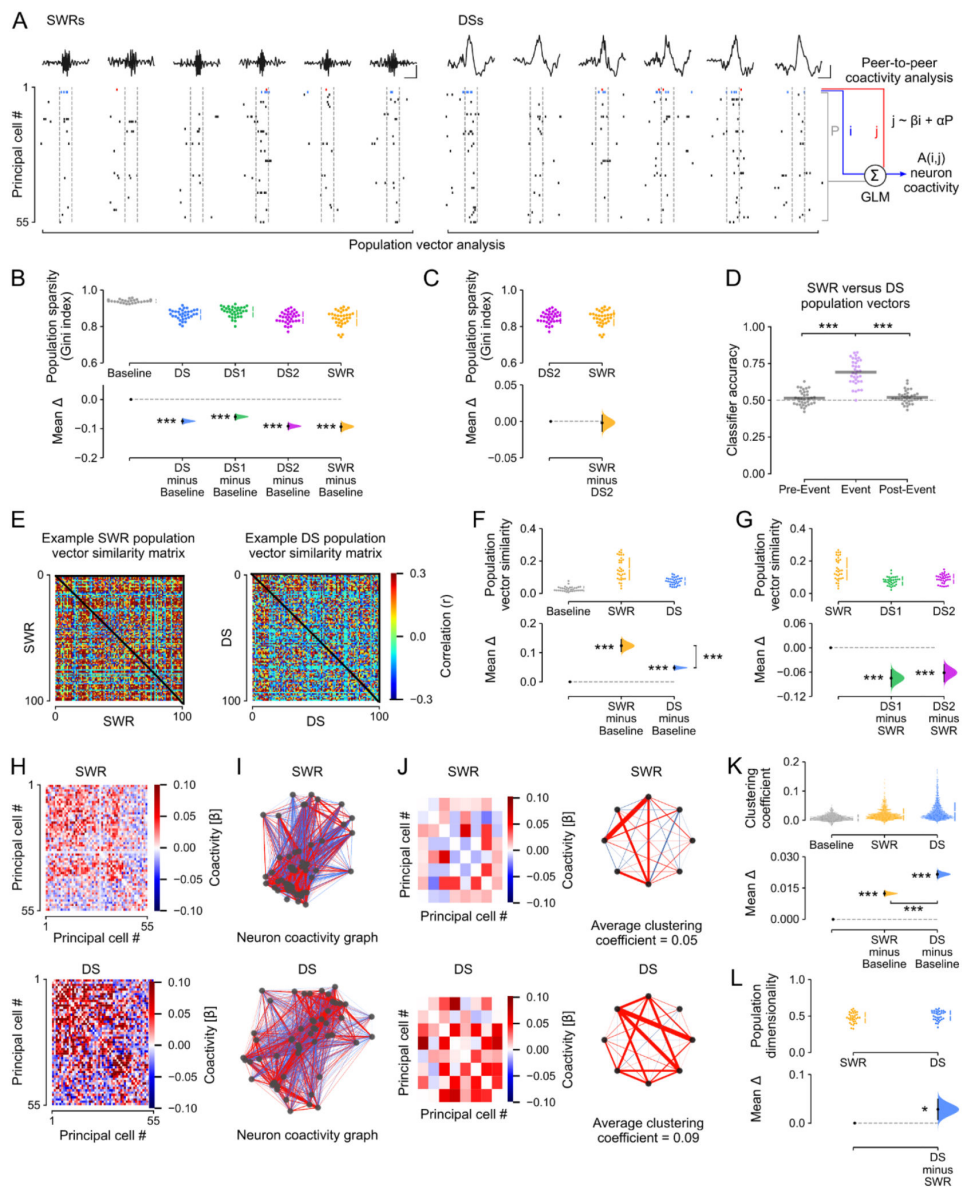
The coactivity structure of population spiking differs between DSs and SWRs. **(A)** Analytical framework: the population-level coactivity structure was analyzed using population vectors of principal cell spiking transiently nested in individual SWRs, DSs, or duration-matched (50 ms) baseline control windows. Scale bars show 20ms and 0.5mV for SWRs and 1mv for DSs. For the analyses in panels B-G, these population firing vectors were then binarized (for each cell: a non-zero spike count gives 1; or else 0). For the analyses in H-K, we calculated the peer-to-peer coactivity, controlling for the overall population activity. **(B, C)** Estimation plots showing the effect size for the differences in population sparsity (using the Gini index) during DSs (with DS_1_ and DS_2_ plotted altogether or separately), SWRs, and compared to equivalent (50 ms duration matched) baseline windows (Baseline) in which no DSs or SWRs occurred. Upper: raw data points (each point represents one session with at least 100 of each event type and 20 principal cells), with the gapped lines on the right as mean (gap) ± s.d. (vertical ends) for each event. Lower: difference (Δ) in sparsity between Baseline windows and all DS, DS_1_, DS_2_, and SWR events computed from 5,000 bootstrapped resamples and with the difference-axis origin (dashed line) aligned to the baseline sparsity (black dot, mean; black ticks, 95% confidence interval; filled curve, sampling-error distribution). (C) as B but comparing population sparsity during SWR versus DS_2_. Note that DS_2_ and SWR events have equivalent sparsity, indicating they engage similar levels of neuronal activity. **(D)** A logistic regression classifier trained on population vectors nested in SWR versus DS events, or matched duration pre-event and post-event control windows, using a 4-fold cross-validation approach (75% of vectors for training; the remaining 25% for evaluation), significantly discriminated DSs from SWRs, but could not discriminate between pre-DS versus pre-SWR, and post-DS versus post-SWR vectors. Gray horizontal bars: mean classification accuracy. **(E-G)** DS population firing vectors are more diverse than those in SWRs. For each sleep session, we computed the similarity (Pearson correlation coefficient) for each pair of population vectors nested in either DSs, SWRs, or duration-matched baseline windows (Baseline). (E) shows example DS and SWR matrices of cross-vector similarity for one session. Cross-population vector similarity was significantly higher in SWRs compared to both DSs and control windows (F), and when compared to DS_1_ and DS_2_ separately (G). **(H-K)** DS and SWR population firing vectors exhibit distinct topology of neuronal coactivity. The coactivity between any two (*i, j*) neurons was measured using a GLM that quantified their short timescale (50 ms windows centered on DS or SWR peaks) firing relationship while accounting for network-level modulation reported by the remaining principal cells in the population (A). (H) This procedure returned for both DS and SWR events an adjacency matrix of *β* regression weights that represented the neuron pairwise coactivity structure of the population (example matrix from one session). (I) Visualization of the corresponding matrices representing DS and SWR based neuronal coactivity graphs. For clarity, (J) shows an example subset (left) for each adjacency matrices shown in (H), along with its corresponding motifs of neuronal coactivity and average clustering coefficient (right). (K) Note that DS-based graphs contained stronger triads of coactive nodes compared to both SWR graphs and control graphs constructed from duration-matched baseline windows (Baseline), as indicated by higher mean clustering coefficients. Each point in the upper plot of K represents the mean clustering coefficient for one hippocampal principal cell (n=1265 neurons, 8 mice) **(L)** The dimensionality of population vector matrices (number of principal components required to explain 90% of the variance) was higher for DSs than SWRs. For B-D, F-G, L: each data point shows one recording session (n=34 recording sessions from 8 mice). The test statistic is the mean difference, shown on the y-axis of each lower plot. P-values are from paired permutation tests, event versus baseline (B,F,K); event versus event (C,F,K,L); or event versus pre-event, event versus post-event (D), *P < 0.05, **P < 0.01, ***P < 0.001.

We next investigated how the hippocampus organizes the collective activity of its principal cells both within individual DS events and across events, comparing these population-level patterns to those expressed in SWRs. To proceed, we first considered the neuron-wise vectors formed by the instantaneous spike discharge of principal cells in DSs, SWRs, or duration-matched control windows (without any DSs or SWRs) of the same sleep/rest (Figures 3A and S4A; “population vector analysis”). This was conducted for all sleep/rest epochs (both those recorded before and those after active exploratory behavior). Using the Gini index ^33,34^, we noted a marked decrease of the mean population sparsity in the spiking vectors nested in DSs and SWRs compared to baseline periods of duration-matched control windows (Figure 3B), with equivalent population sparsity levels during DS_2_ versus SWRs (Figures 3C and S4B-C). A logistic regression classifier trained on a subset of these population firing vectors and iteratively tested on the remaining subset significantly distinguished between DS and SWR events but could not distinguish between their corresponding pre-event nor their post-event control epochs (Figure 3D). Successful classification was also obtained when using only DS_2_ events, which matched SWRs in the mean population sparsity per event (Figure S4D). When evaluating the pairwise similarity of DS-nested population vectors versus those of SWR-nested vectors (Figure 3E), we further observed that DSs contained a higher diversity (i.e., lower similarity) of firing vectors compared to those in SWRs, which in turn were more similar to one another (Figures 3F,G and S4E-H).

This difference in population vector similarity suggested that DSs and SWRs differ with respect to their neuronal motifs of transient coactivation. By examining the topological organization of peer-to-peer firing associations, we indeed observed that DS events contain stronger motifs of coactive principal cells than SWRs. For each cell pair (*i, j*), we trained a generalized linear model to predict the spike discharge of neuron *j* from that of neuron *i* while accounting for the activity of the remaining peers (Figure 3A; “peer-to-peer coactivity analysis”). We performed this procedure separately for DS and SWR events, which returned for each type of network event a matrix of *β* regression weights that represented the coactivity structure of the population (Figure 3H). With these matrices, for both DS and SWR events we constructed neuronal coactivity graphs (with no self-connections) where each node is a cell and the edge linking any two nodes represents the firing association of that cell pair (Figure 3I,J). This revealed that DS-based graphs contained stronger triads of coactive nodes compared to SWR graphs (Figures 3K and S4I,J). This remained the case when directly comparing DS_2_ and SWR events (Figure S4K), and when calculating the neuron-wise average coactivity strength (Figure S4L).

These findings showed that while both DS and SWR events synchronize hippocampal principal cells, population coactivity responses to DSs are more diverse. To further assess this, we applied principal component analysis to quantify and compare the variance explained by the activity patterns nested in DS_1_, DS_2_, and SWR. This revealed higher dimensionality of population vectors in DS events compared to SWRs (Figures 3L and Table S1). This was accounted for by DS_2_ firing vectors, with those nested in DS_1_ requiring fewer principal components to explain equivalent variance and exhibiting lower dimensionality than those in DS_2_ and SWRs (Figure S4M-P).

### Waking theta coactivity patterns reactivate in offline DSs and support flexible memory

**Figure 4:**
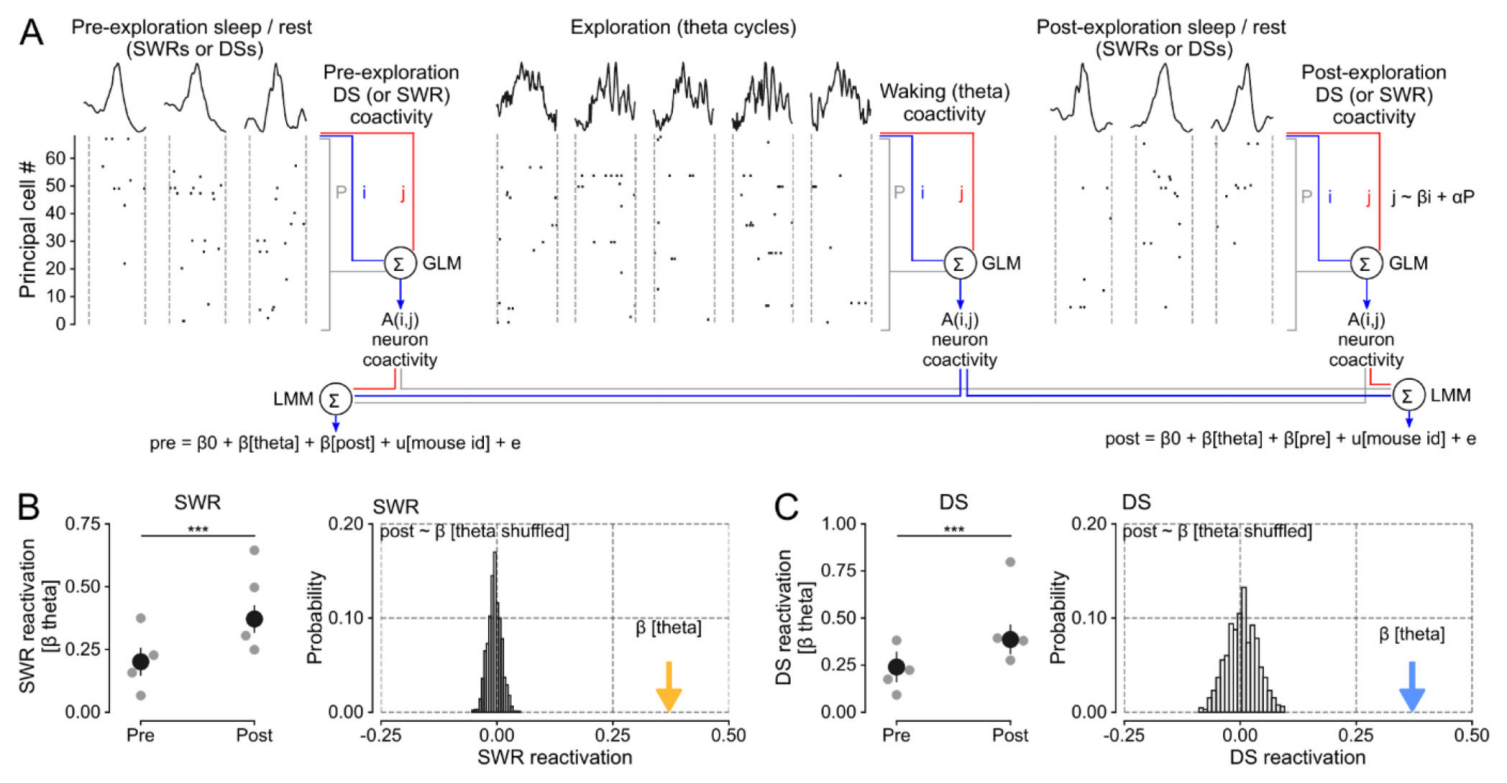
Waking patterns of hippocampal coactivity reactivate in offline DSs. **(A)** DS and SWR reactivation of waking patterns formed by principal cell theta coactivity. For each cell pair (*i, j*), we predicted the spike discharge of neuron *j* from the activity of neuron *i* while regressing out the activity of the remaining population during pre-exploration sleep, exploration of open-field arenas, and post-exploration sleep (using GLMs as in Figure 3A). We separately applied this procedure for DSs and SWRs in both sleep/rest sessions (offline DS versus offline SWR coactivity), and across theta cycles in the exploration session (waking theta coactivity). This procedure returned a matrix of *β* regression weights that represented the neurons pairwise coactivity structure of the population in each session. We then used a Linear Mixed Model (LMM) to compare the waking theta coactivity with post-exploration sleep coactivity (in DSs or SWRs) while controlling for pre-exploration sleep coactivity (in DSs or SWRs), and vice versa (reverse model). We included mouse identity as a random factor in each LMM. **(B)** SWR reactivation (measured by the β coefficients of the LMM that predicted post-exploration SWR coactivity from waking theta coactivity, controlling for pre-exploration SWR coactivity). Left: The β coefficient for theta coactivity was significantly higher when predicting post-exploration SWR coactivity than with the reverse model (i.e., predicting pre-exploration SWR coactivity from theta coactivity, controlling for post-exploration SWR coactivity). Gray points show the β coefficient for theta coactivity for individual mice. Error bars show ± 95% confidence interval. P-value from t-test comparing post versus pre β coefficients: t(7308) = 10.29; *P* < 0.0001. Right: The histogram shows the random probability distribution of β weights for theta coactivity when cell pair identity was shuffled (i.e., a null distribution based on 1,000 random shuffles; n=7,310 cell pairs from 4 mice). The colored arrow shows the actual β coefficient for theta coactivity. **(C)** DS reactivation exhibited the same pattern of results as SWR reactivation, shown in B. P-value from t-test comparing post versus pre β coefficients t(7308) = 8.84; *P* < 0.0001.

The DS-nested motifs of peer-to-peer firing associations could instantiate population patterns of neuronal coactivity undergoing offline reactivation to support memory-guided behavior. Notably, the link between hippocampal SWRs and memory reactivation was initially established through the observation that the neural patterns of joint spiking activity expressed during exploratory behavior are more strongly correlated with those nested in post-exploration sleep/rest SWRs than those in SWRs before waking experience ^8,13^. Accordingly, we next determined whether DSs constitute another hippocampal timeframe for offline reactivation of waking coactivity patterns. To proceed, we used our peer-to-peer coactivity analysis (Figure 3A), applying it to DS versus SWR events of sleep/rest before and after exploration of open field arenas (Figures 4A and S5). Likewise, we obtained the waking patterns of population coactivity in theta cycles during exploration. With these, we computed DS and SWR reactivation by measuring the tendency of the peer-to-peer theta firing associations to reoccur in post-exploration sleep/rest DS (or SWR) events, while controlling for prior pre-exploration DS (or SWR) coactivity and mouse identity, using a linear mixed model. In line with previous work, offline patterns of SWR coactivity reflected those of theta coactivity significantly more during post-exploration than pre-exploration sleep/rest (Figure 4B, *left panel*; Figure S5B,D). This SWR reactivation was significantly higher than that obtained with a null distribution generated from models using randomly shuffled cell pair identities (Figure 4B, *right panel*). Importantly, we observed that theta coactivity patterns were also strongly reactivated in post-exploration DSs (Figures 4C, S5C,E and Table S2). The clustering coefficient was higher in DSs than SWRs, and also increased from pre- to post-exploration sleep in both SWRs and DSs (Figure S5F,G). Analyses of DS_1_ and DS_2_ separately showed evidence for reactivation during both types of post-exploration DS events (Figure S5H,I). By applying the same analytical framework to the neuronal ensembles tracked in SWRs and DSs, this result provided evidence for offline DS reactivation of hippocampal waking firing patterns.

**Figure 5:**
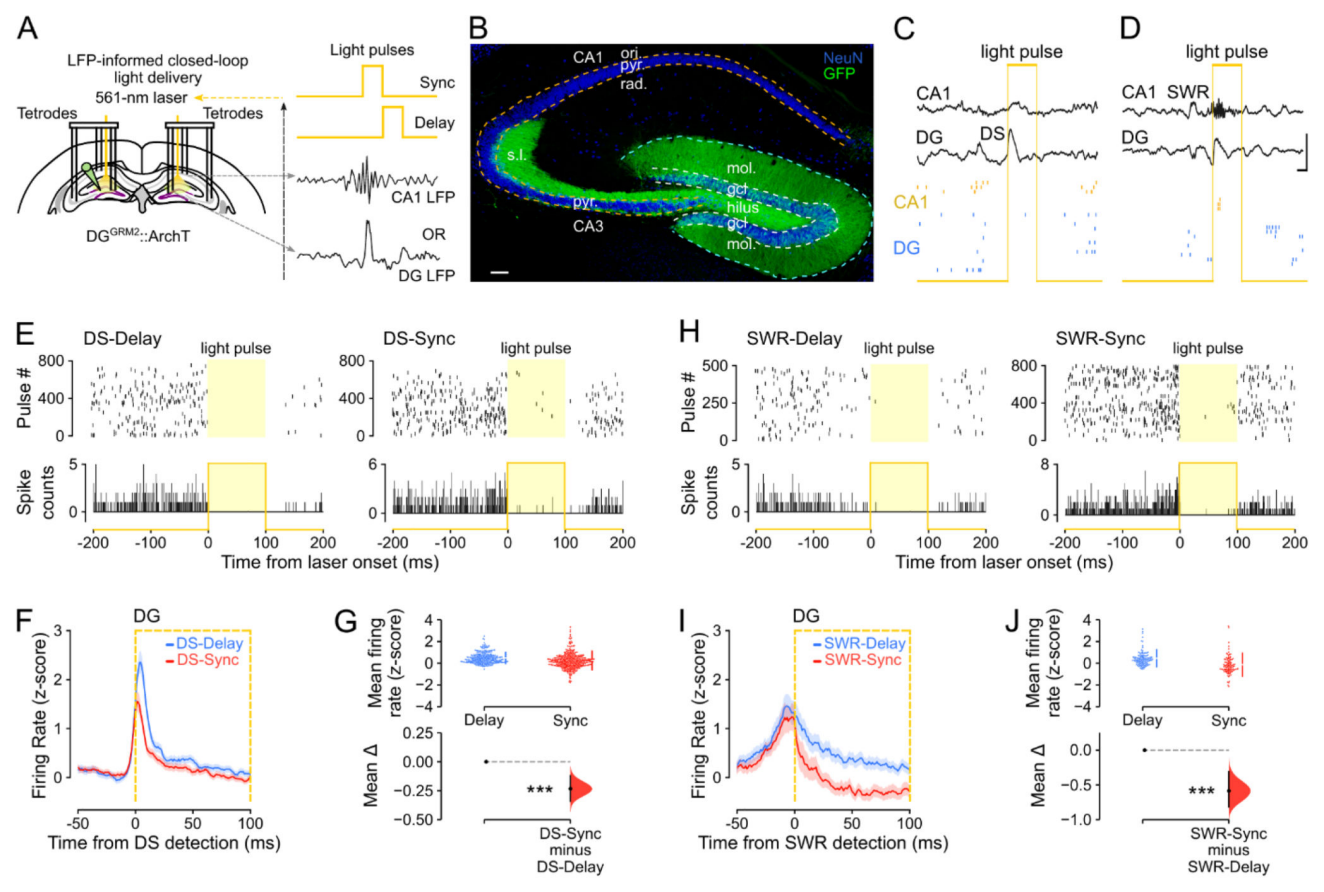
DS- and SWR-informed offline suppressions of DG granule cell activity. **(A)** Triple-(DG-CA3-CA1) ensemble recording with LFP-informed yellow (561 nm) DG light-delivery. Dentate granule cells (DGCs) transduced with ArchT-GFP (DG^Grm2^::ArchT). Closed-loop light-delivery to suppress DGC spiking immediately upon either DS detection (DS-Sync condition) or SWR detection (SWR-Sync) or their respective control conditions (DS-Delay and SWR-Delay, where light delivery was offset by 100 ms after event detection). **(B)** ArchT-GFP-expressing DGCs in a DG^Grm2^::ArchT mouse. Neuronal nuclei stained with NeuN. Scale bar=100 μm. Granule cell layer: gcl; molecular layer: mol; pyramidal cell layer: pyr; stratum oriens: ori; radiatum: rad; lucidum: s.l. **(C, D)** Closed-loop feedback transiently silenced DGCs during either DG DS (C; “DS-Sync”) or CA1 SWR (D; “SWR-Sync”) events, illustrated with raw data examples. Scale bars, 30 ms (horizontal), 1.5 mV (vertical). **(E)** Raster plots (event-related spiking response; one light pulse per row (Upper), and peri-event time histograms (Lower) showing photo-silencing of two example DG cells from a DG^Grm2^::ArchT mouse in DS-Delay and DS-Sync. **(F, G)** Corresponding quantification of average DGC firing rate (z-score) for DS-Delay versus DS-Sync (F,G; n=548 cells in 9 mice). In F, the orange box shows the laser-on period for DS-Sync. **(H-J)** As E-G but showing DGC photo-silencing during SWR-Delay and SWR-Sync conditions (I, J; n=181 cells in 3 mice). In I, the orange box shows the laser-on period for SWR-Sync. For G and J, the test statistic is the mean difference, shown on the y-axis of each lower plot. P-values are from unpaired permutation tests, Delay versus Sync, ***P < 0.001.

The offline reactivation of waking population patterns in sleep/rest DSs (Figure 4C), which contain more diverse and higher-dimensional patterns of neuronal coactivation than those found in SWRs (Figure 3F,L), raised the question of their network contribution to memory-guided behavior. We thus tested whether the offline population response during DSs was necessary to perform tasks that require integrating multiple items in memory to flexibly distinguish between familiar and novel stimuli. To this end, we transduced DG granule cells with the yellow (561-nm) light-driven optogenetic silencer Archaerhodopsin T (ArchT) in Grm2-Cre mice (Figure 5A,B). We then implanted these DG^Grm2^::ArchT mice for triple-(DG-CA3-CA1) ensemble recordings combined with bilateral optic fibers for DG light delivery. In these experiments, DG light delivery was performed in a closed-loop manner during sleep/rest using the real time detection of either DSs (in the DG LFPs), or SWRs (in the CA1 LFPs; Figure 5A,C,D; “DS-Sync” or “SWR-Sync” conditions). We also used a within-subject control paradigm whereby, on different days, light was not synchronized to but instead delivered after each DS or SWR had elapsed (“DS-Delay” or “SWR-Delay” conditions). DS synchronized light delivery did not affect the amplitude of the DG or CA1 LFPs, nor CA1 ripple duration, occurrence probability, or power (Figure S6A-I). DS-synchronized light delivery significantly reduced spiking activity in DG neurons compared to when laser-onset was DS-delayed (Figure 5E-G); and also reduced spiking activity in CA principal cells (Figure S6J,K). Paired analysis of the firing rates of dentate granule cells during DS events without light delivery versus those with light deliver (DS-Sync) also showed significantly reduced instantaneous spiking (15.0±0.7 versus 10.4±0.6 Hz). SWR-synchronized light delivery also significantly suppressed DG neuronal spiking (Figure 5H-J).

**Figure 6:**
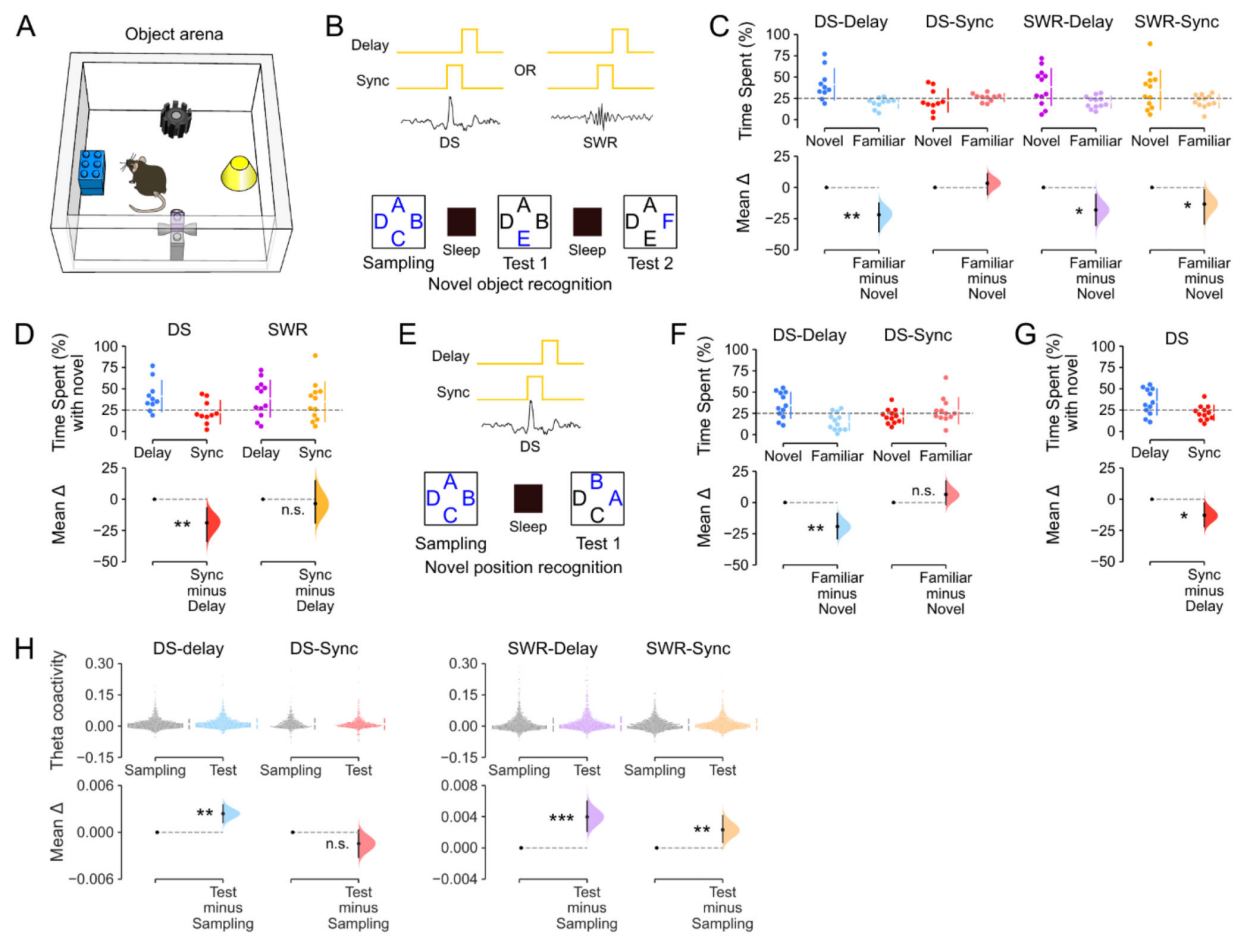
Offline suppression of DS activity impairs flexible recognition memory. **(A)** Behavioral arena used for the recognition memory tasks. **(B-D)** Offline DS events are required for novel object recognition memory. (B) Task layout. During sleep sessions (interposed between novel object exploration sessions), closed-loop optogenetic suppression of DG cells in DG^Grm2^::ArchT mice was achieved using real-time monitoring of either DG or CA1 LFPs to actuate either DS synchronized (DS-Sync) or delayed (DS-Delay), SWR synchronized (SWR-Sync) or delayed (SWR-Delay) DG light delivery. Letters depict object locations in the task arena (A), with novel objects in blue. (C) Estimation plot showing the percentage of time spent by mice with the novel versus the familiar objects in each of the four LFP-informed closed-loop conditions. Upper: Each data point represents the percentage time spent with the novel object versus the mean percentage time spent with the three familiar objects; chance performance is shown by the dashed line. Lower: mean difference between novel and familiar object exploration time. (D) as C but directly comparing novel object preference in the delay versus sync conditions for DS and SWR events. Mice in the DS-Delay, SWR-Delay, and SWR-Sync conditions, but not the DS-Sync condition, exhibited a significant preference for novel over familiar objects (DS-Delay and DS-Sync: n=10 sessions, in 3 mice; SWR-Delay and SWR-Sync: n=12 sessions in 3 mice). **(E-G)** Likewise, offline DS events are required for novel position recognition memory. (E) Task layout. Letters depict object locations, with novel positions in blue. (F) Estimation plot showing the percentage of time spent by DG^Grm2^::ArchT mice with the novel versus the familiar object locations following sleep sessions with DS-Sync or DS-Delay suppression of DG cells. Upper: Each data point represents the percentage time spent with objects in novel locations versus objects in familiar locations; chance performance is shown by the dashed line. Lower: mean difference between novel and familiar location exploration times. (G) As F but directly comparing novel location preference in DS-Delay versus DS-Sync. Mice in the DS-Delay but not the DS-Sync condition exhibited a significant preference for objects in novel over familiar locations (n=12 novel versus n=12 familiar locations, 6 sessions, in 4 mice). **(H)** In the object recognition task, the theta peer-to-peer coactivity increased from the initial object sampling to the memory test following offline DG cell suppression in the DS-Delay, SWR-Delay, and SWR-Sync conditions; but this was not the case in the DS-sync condition (where mice exhibited no novel object preference). Paired estimation plot showing theta coactivity during Sampling versus Test. Upper: each point represents a beta coefficient for the theta-nested peer-to-peer coactivity between pairs of hippocampal principal cells (n=1537, n=678, n=1719, n=1482 cell pairs, respectively, in 6 mice). Lower: black dot, mean difference between sampling and test sessions; black ticks, 95% confidence interval. For C,D and F-H, the test statistic is the mean difference, shown on the y-axis of each lower plot. P-values are from paired permutation tests, Familiar versus Novel (C,F); Delay versus Sync (D,G); or Test versus Sampling (H), *P < 0.05, **P < 0.01, ***P < 0.001.

We applied these closed-loop light-delivery approaches during interposed sleep/rest sessions in three behavioral tasks (Figures 6 and S6L-T). The first, hippocampal-dependent task required mice to recognize previously encountered (familiar) versus novel objects ^35^. In this novel-object recognition task, mice repeatedly explored a square-walled arena containing four objects (Figure 6A,B). In the first session (‘Sampling’), mice encountered four distinct novel objects, each one placed beside a wall. On the subsequent sessions (‘Test’), one of the initially sampled objects was replaced by a different novel object so that the mouse could explore one completely novel object along with the three ‘familiar’ objects from the previous session that day. These exploration (sampling and test) sessions alternated with sleep/rest sessions where mice received DG-targeted light delivery, either synchronized or delayed with respect to either DS or SWR onset, thus yielding four distinct experimental conditions (DS-Delay, DS-Sync, SWR-Delay, SWR-Sync). In each test session *n*, we measured novelty preference using the proportion of time mice spent investigating the novel versus the familiar objects, thereby probing recognition memory for session *n* − 1. We found that novelty detection was not impaired in test sessions following sleep with DG granule cell suppression in either DS delayed, SWR delayed, or SWR synchronized conditions: mice subsequently expressed a stronger preference for novel over familiar objects under these three conditions (Figure 6C,D). This novel object preference was similar to that observed in control mice without any optogenetic intervention (Figure S6L). However, novel object preference was absent in test sessions following DS-synchronized suppression (Figure 6C,D). The total object exploration time, number of laser pulses and number of SWRs did not differ between the DS-synchronized and DS-delayed conditions (Figure S6M-O).

We also tested the offline DS-informed suppression of DG granule cells after tone fear conditioning as a non-hippocampal-dependent task ^36^. Mice were trained with five tone-shock pairings and, following DS-synchronized or DS-delayed suppression, we evaluated fear memory by measuring freezing behavior during a recall session in which tones were played but no shocks were given. Compared to baseline freezing (measured during the first tone of training, before any shocks were given), mice exhibited higher (and equivalent) freezing levels during recall regardless of whether they had received DS-synchronized or DS-delayed suppression (Figure S6P-S).

We finally tested whether DS-synchronized suppression affected performance in a novel-position recognition task that is reportedly more sensitive to DG than CA1 lesions, whereas novel-object recognition requires both DG and CA1 ^37^. This novel-position task is similar to the novel-object recognition task in that mice explore four novel objects during the sampling phase (Figure 6E). However, rather than introducing a new object in the test phase, the locations of two of the initially sampled objects are swapped for the subsequent session, leaving the other two objects in their original locations (Figure 6E). We found that mice preferentially explored the novel-positioned objects following DS-delayed suppression of DG granule cells but showed no such preference following DS-synchronized suppression (Figures 6F,G and S6T).

Recent work has identified that the continued integration of new items in memory is associated with increased neuronal coactivity patterns nested in hippocampal theta oscillations ^38^. In line with this, we found that the preserved object recognition memory observed after offline DG cell suppression in the DS-delayed, SWR-delayed, and SWR-synchronized conditions was accompanied by stronger theta coactivity (Figure 6H). This was not the case following DS-synchronized suppression (Figure 6H), indicating that DS silencing disrupts the integration of recently experienced information. Collectively, these results show that the hippocampal population response to offline DS events is required for flexible, memory-based recognition of previously encountered items and associated network gain in theta coactivity.

## Discussion

Our findings establish that offline DSs activate neurons across the DG and CA regions. DSs are therefore a second hippocampal network event that hosts short timescale coactivation forming population-level neural patterns, like the well-established SWRs. However, the activity structure and neuronal content are distinct in DSs. Notably, we found that DSs nest stronger motifs of coactive neurons, yielding population patterns of higher diversity and dimensionality compared to those in SWRs. Like SWRs, DSs reactivate hippocampal population patterns expressed in prior waking experience. This offline reactivation is behaviorally significant: closed-loop suppression of DG granule cell spiking selectively during offline DS events is sufficient to disrupt downstream CA principal cell activity and impair flexible recognition memory for previously encountered items, as well as the associated network gain in theta-nested neuronal coactivity. Collectively, these findings identify a core contribution for DSs to hippocampal patterns of population activity and memory-guided behavior.

We started this investigation by observing that DSs increase spiking activity in principal cells across the DG, CA3, and CA1 regions of the hippocampus. This finding is consistent with previous reports of DS-evoked spiking activity in DG granule cells but contrasts with some earlier reports of DS-suppressed CA pyramidal cell spiking ^22,24–26^. Notably, Bragin and colleagues reported suppressed spiking in 3/14 CA3 principal cells and suppressed CA1 multi-unit activity in 2/10 rats, showing some, rather than consistent, CA suppression. In addition, Penttonen et al. reported suppressed CA1 multi-unit activity and hyperpolarization of 4 intracellularly recorded CA1 neurons during DSs in anaesthetized rats. However, DS rates are ~10-fold lower and have smaller amplitude during anesthesia compared to DSs observed during natural sleep/rest ^25^. Other studies reported increased CA1 multi-unit activity ^26^ and increased CA3 single-unit spiking during DS events ^24^ in non-anaesthetized mice. Here, we report a variety of firing responses across individual CA neurons, ranging from strong activation to suppression during DS events (Figures 1G and 2F). However, our systematic study (including > 3,500 principal cells; Supplemental Table S3) shows that DSs do indeed drive increased mean population spiking activity in both DG and CA principal cells (Figure 2G).

Previous studies distinguished between two types of DS event (DS_1_ and DS_2_), based on the laminar profile of their transmembrane currents ^22,24,26^. Here, we found that DS_2_ are more effective than DS_1_ events at recruiting hippocampal principal cells, with higher spike rates per cell and more coactive cells per event. This result is consistent with a recent report that DS_2_ but not DS_1_ events reliably increase spiking in DG and CA3 principal cells ^24^. The same report saw only a slight increase in CA1 spiking during DS_2_, and no effect of DS_1_ events on either CA1 or CA3 principal cells. They also found that DG principal cell spiking was suppressed during DS_1_, in contrast to our study and previous reports ^22^. Here we found that both DS_1_ and DS_2_ events evoked significantly increased spiking activity in DG, CA3, and CA1 principal cells, but again would emphasize the diversity of CA cell responses, especially during DS_1_ events (Figure 2F). The observed differences between studies might reflect differences between DS events in head-fixed awake mice versus those in sleep/rest ^22,29^. They could also indicate a moment-to-moment diversity across individual DSs, similar to that highlighted for individual SWRs ^39^ and theta cycles ^40^.

In this study, we directly compared patterns of population spiking activity in DSs versus SWRs. We found that population firing differed across these two types of network event; allowing a classifier to distinguish DSs versus SWRs based on their instantaneous vectors of principal cell spiking (Figure 3D). We also observed that population patterns in DSs are overall more diverse (less correlated) than those in SWRs, showing stronger triads of coactive neurons and higher dimensionality (Figures 3 and S4). DS_1_ and DS_2_ population patterns yet showed distinct trends with respect to SWRs: DS_1_ firing vectors exhibited less diversity (i.e., required fewer principal components to explain most of the variance; Figure S4O) and lower dimensionality (Figure S4P) than those in SWRs; DS_2_ firing vectors showed the opposite trend. Previous work has reported that DS_1_ and DS_2_ events relate to distinct entorhinal cortex inputs, with DS_1_ relying more on lateral entorhinal cortex while DS_2_ events rely more on the medial entorhinal cortex ^24^. This suggests a possible division of mnemonic labor where DS_1_ population patterns would favor non-spatial information streams while DS_2_ might favor spatial information ^41^. We also found that waking patterns of neuronal coactivity nested in theta oscillations reactivate in DSs of post-exploration sleep/rest (Figures 4 and S5). Notably, the distributions of coactivity values in DSs indicate both positive and negative firing associations (Figure S5E). The coexistence of correlated and anti-correlated spiking activities in DSs could reflect a Hebbian learning rule as reported in SWRs ^42^ whereby positive and negative changes in hippocampal principal cell firing associations can shape offline DS reactivation as a function of recent waking experience. These findings provide important evidence for offline reactivation of hippocampal waking firing patterns outside of SWRs, stimulating new avenues for future work to explore.

To determine whether spiking activity observed during DS events was required for subsequent memory-guided behavior, we deployed a closed-loop optogenetic feedback approach to suppress DG granule cell activity selectively during DS events (Figures 5 and S6). Real-time inhibition of the DG in Grm2-Cre mice did not yield a complete suppression of the spiking activity in dentate granule cells. This also did not alter the magnitude of dentate spikes, which powerful expression in the DG LFPs could reflect the high cellular density of the granule cell layer and its strong neural inputs. This DS-synchronized suppression of DG principal cells reduced concomitant spiking activity in CA principal cells, but did not affect the expression of SWRs in CA1. When applied in sleep/rest following object-location exploration, this DS-synchronized neural suppression impaired subsequent memory performance in both novel-object and novel-position recognition tasks. Although ours is the first study to leverage a closed-loop optogenetic approach, these findings are consistent with previous behavioral studies using electrical stimulation to disrupt hippocampal activity during DSs ^32,43,44^. While both approaches provide strong evidence for a central contribution of DS events in memory-guided behavior, it is important to recognize that optogenetic and electrical interventions do not recapitulate natural hippocampal activity patterns. Moreover, DS_2_ represents the dominant type of DS event that exhibit, in comparison to DS_1_, stronger firing rate increase of DG, CA3, and CA1 principal cells (Figure 2G), neuronal recruitment (Figures S3E,F) and coactivity (Figure S4L) and lower dimensionality (Figure S4O,P). The major effect of DS activity on memory may be associated with DS_2_, a hypothesis that future technological (closed-loop controller) development for differential manipulation of DS_1_ versus DS_2_ would be able to test. We also found that increased theta coactivity was associated with recognition memory, and that this network gain in theta coactivity was absent following DS-synchronized neural suppression (Figure 6H). This finding is consistent with recent work showing that continual integration of new memory items across behavioral experiences increases neuronal coactivity ^38^. Altogether, these results support the idea that neuronal activity during DS events plays an important role in subsequent memory-guided behavior, as SWRs do.

Why does the hippocampus use more than one offline network mechanism to support memory? DSs and SWRs are driven by distinct neural circuits. SWRs depend on excitatory inputs from CA3 to the CA1 stratum radiatum, generating high-frequency ripples in the CA1 pyramidal layer ^7,45–47^. DSs are non-oscillatory events associated with excitatory inputs from the entorhinal cortex to the DG molecular layers ^22,24,29^. Notably, entorhinal cortex lesions eliminate DSs but increase SWR incidence ^22^. Our structural analysis of DS versus SWR population patterns raises the intriguing possibility that SWRs may be more suited for lower-dimensional network coactivity serving robust information flow; whereas DSs may promote higher-dimensional activity, allowing diverse mnemonic patterns to coexist offline and support flexible, pattern separation for subsequent behavior. Collectively, these findings open important new avenues for future work to explore the interplay between DS versus SWR events as two distinct timeframes for the hippocampus to optimize offline computations serving memory-guided behavior.

## STAR Methods

### Resource Availability

#### Lead Contact

Further information and requests for resources and reagents should be directed to and will be fulfilled by the Lead Contact, David Dupret (david.dupret@bndu.ox.ac.uk).

#### Materials Availability

This study did not generate new unique reagents

### Experimental Model and Study Participant Details

#### Animals

These experiments used adult (4–6 months old) wild-type C57Bl6/J mice (Charles River Laboratories, Kent, UK) or hemizygous Nestin-Cre mice (Jackson Laboratories; B6.Cg-Tg(Nes-Cre)1Kln/J, stock no. 003771, RRID: IMSR_JAX:003771) for the initial investigation of principal cell spiking in DSs and SWRs using tetrodes or silicon-probe recordings (Figures 1–4). To optogenetically target DG granule cells, we used adult metabotropic-glutamate-receptor 2-Cre (Grm2-Cre) hemizygous male mice (Figures 5, 6). This Grm2-Cre mouse strain was obtained from the Mutant Mouse Resource and Research Center (MMRRC; Tg(Grm2-cre)MR90Gsat/Mmucd; stock no. 034611-UCD, RRID:MMRRC_034611-UCD) at University of California at Davis, an NIH-funded strain repository, and was donated to the MMRRC by Nathaniel Heintz, Ph.D., The Rockefeller University, GENSAT and Charles Gerfen, Ph.D., National Institutes of Health, National Institute of Mental Health. All mice were group housed with same-sex littermates until the start of the experiment and singly housed after surgery. Mice had free access to food and water throughout, in a dedicated housing room with a 12/12 h light/dark cycle (7 a.m.–7 p.m.), 19–23 °C ambient temperature and 40–70 % humidity. This study used mice with good health/immune status, that were not involved in previous procedures, and were drug and test naïve at the start of the experiments. Mice were adult males and the influence (or association) of age and sex, or both on the results of the study was not tested. This represents a limitation to this research’s generalizability. All experiments were performed between 8 a.m.–6 p.m. during the light-on period, that is when mice sleep more. Experiments were performed in accordance with the Animals (Scientific Procedures) Act, 1986 (United Kingdom), with final ethical review by the Animals in Science Regulation Unit of the UK Home Office.

### Method Details

#### Viral vectors

An AAV carrying a double-floxed inverse open reading frame (DIO) Cre-dependent opsin under the CAG promoter was used to deliver Archaerhodopsin (ArchT) (Han *et al*., 2011) into DG granule cells (AAV9-CAG-Flex-ArchT-GFP, titer: 8.3 × 10^12^ TU / mL, University of North Carolina).

#### Surgical procedures

Mice received viral injections and microdrive implantations under gaseous isoflurane anaesthesia (~1% in 1 L / min O_2_), with systemic and local analgesia administered subcutaneously (meloxicam 5 mg / kg; buprenorphine 0.1 mg / kg; bupivacaine 2 mg / kg). Viruses were injected bilaterally into the dorsal DG (3 × 200 nL per hemisphere; at the following stereotaxic coordinates from bregma: anterior-posterior: -1.6, -2.4, -2.4; mediolateral: ±1.0, ±1.2, ±1.5; dorsoventral: -1.7, -1.7, -1.7 mm, respectively), and delivered using a pulled glass micropipette (~16 μm i.d.) at a rate of 100 nL min^−1^, with an additional 100 nL min^−1^ diffusion time with the pipette *in situ*. In a separate surgery, mice were implanted with a microdrive containing twelve- or fourteen-independently movable tetrodes bilaterally targeting DG, CA3, and CA1, and two optic fibers (Doric Lenses Inc., Quebec, Canada) positioned bilaterally above the dorsal DG. Tetrodes were constructed by twisting together four insulated tungsten wires (12.7 μm diameter, California Fine Wire, CA, USA) which were briefly heated to bind them together into a single bundle. Each tetrode was loaded in one cannula attached to a 6 mm long M1.0 screw to enable its independent depth manipulation. A separate group of mice were implanted with unilateral single-shank 64-channel silicon-probe (model: ASSY-236 H3, 8 mm; Cambridge Neurotech, Cambridge, UK; stereotaxic coordinates from bregma: anterior-posterior: -2.0; mediolateral: -1.7 mm); these mice did not receive prior viral injections.

#### Recording procedures

Following the implantation surgery, mice recovered for at least seven days before familiarization to the recording procedure. Mice were handled daily and exposed to the sleep-box for > 0.5 h per day for at least four days. During this period, tetrodes / silicon-probes were slowly lowered to the proximity of the cell layers. Once at the correct depth, silicon-probes were left in the same position for the rest of the experiment. Tetrodes were lowered into the CA1, CA3 pyramidal or DG granule cell layers on the morning of each recording day in search of multi-unit spiking activity, using the electrophysiological profile of the local field potentials including sharp-wave ripples, gamma oscillations, and dentate spikes to further guide placement. Tetrodes were left in position for ~1.5–2 h before recordings began on that day. At the end of each recording day, tetrodes were raised (~150 μm) to protect hippocampal the cell layers from potential mechanical damage overnight. We lowered again each individual tetrode on the next morning in search of cells, making it unlikely that the recorded units are the same neurons across days. During recording sessions, mice explored open-field environments (41 cm diameter cylinder, or 41 × 41 cm square box, both with 30 cm high walls), or were placed in a sleep box containing sawdust bedding and nesting material (12 × 12 × 28 cm, length × width × height). The instantaneous speed and the theta-to-delta ratio profiles for DS and SWR events corresponded to those of sleep (Figure S1G,H). However, in the absence of electromyography signals or other additional signals in defining a sleep stage, we here refer to sleep/rest. Each open-field or sleep box recording session lasted ~15-30 min. Experiments were performed under dim light conditions (~20 lux) with low-level background noise (~50 dB).

#### Light delivery

A 561 nm diode pumped solid-state laser (Crystal Laser, model CL561-100; distributer: Laser 2000, Ringstead, UK) was used to deliver green-yellow light bilaterally to the dorsal DG (~2-4 mW) via a 2-channel rotary joint (Doric Lenses Inc.).

#### Multichannel data acquisition

Electrode signals were amplified, multiplexed, and digitized using a single integrated circuit (headstage) located on the head of the animal (RHD2164, Intan Technologies, USA; http://intantech.com/products_RHD2000.html). The amplified and filtered (pass band 0.09 Hz to 7.60 kHz) electrophysiological signals were digitized at 20 kHz (RHD2000 Evaluation Board) and saved to disk with the synchronization signals from the positional tracking and laser activation. To track the location of the animal, three LEDs were attached to the headstage and captured at 25 frames per second by an overhead color camera.

#### Spike sorting and unit isolation

Spike sorting and unit isolation were performed via automatic clustering software Kilosort (Pachitariu *et al*., 2016, 2023) (https://github.com/cortex-lab/KiloSort) followed by graphically based manual recombination using cross-channel spike waveforms, auto-correlation histograms and cross-correlation histograms within the SpikeForest framework (https://github.com/flatironinstitute/spikeforest) (Magland *et al*., 2020). All sessions recorded on a given day were concatenated and cluster cut together to monitor cells throughout the day. Each unit used for analyses showed consistent spike waveforms and stable firing rates throughout the entire recording day. Tetrode location in the dorsal-ventral axis for each recording day (Figure S1C) was determined using laminar LFP signatures, as described in detail in Lopes-dos-Santos et. al. (2023), and later confirmed in the *ex vivo* histology (Figure S1B).

#### Principal cell versus interneuron classification

Hippocampal principal cells were distinguished from interneurons by the trough-to-peak width of the spike waveform, as previously described ^48^. Briefly, to evaluate the waveform consistency for each unit, we used the waveform with the maximum amplitude across the tetrode channels for each cluster. We compared the prominence of a unit mean waveform amplitude to the standard deviation stemming from all its spikes by computing a waveform score:

$$wv_{score\;}=\sqrt{\sum_{i=1}^{n}\frac{{\left(w_{i}/\sigma_{i}\right)}^{2}}{n}}$$

where *w*_*i*_ is the value of the mean waveform at sample *i, σ*_*i*_ is the standard deviation at sample *i* across all spikes, and *n* is the number of waveform samples. This metric quantifies the relative magnitude of the mean waveform amplitude against the spike-to-spike variability. Clusters with a waveform score above 0.75 and a refractory period violation below 2% (quantified as the proportion of intervals shorter than 2 ms in the ISI distribution) were included for further analyses. We categorized units as either putative interneurons or principal cells based on the width of their waveform as indicated by the trough-to-peak latency. In a prior dataset of ~4,000 neurons, we noted a bimodal distribution in trough-to-peak latency. Fitting this with a 1-dimensional, 2-component Gaussian Mixture Model (GMM), we set the classification threshold where the two Gaussian components intersect: units with latencies above were labeled as putative principal cells, and those below as putative interneurons. The same inclusion criteria and classification procedures were used for DG, CA3 and CA1 neurons. In total, this study includes 3,619 hippocampal principal cells (CA1, n = 1,322; CA3, n = 573; DG, n = 1,724; from 134 total recording days in 25 mice).

#### Local field potential signals

LFP signals were processed by first applying an anti-aliasing filter (8^th^-order Chebyshev type I filter) to the wide band signals sampled at 20 kHz. These signals were then down-sampled to 1,250 Hz using the decimate function from the signal submodule of Scipy (version 1.11.2).

#### Dentate spike detection

Dentate spikes were detected during sleep sessions from LFPs recorded from tetrodes located in the DG granule cell layer or silicon-probes with recording contacts in the DG granule cell layer. In silicon-probe recordings, we initially subtracted the LFP signals from all channels using a reference channel found in the stratum oriens. LFPs were band-pass filtered (1–200 Hz, using a 4th order Butterworth filter). The mean and standard deviation of the LFP amplitude were calculated across the entire sleep session and peaks that exceeded a threshold of six times the median absolute value of the filtered signals were designated as dentate spikes. The time bin with the largest local maximum was taken as the peak of the dentate spike, and this timestamp was recorded. If more than one peak appeared within a 50 ms frame, we retained only the highest amplitude peak. On recording days with several tetrodes in the DG, we used the tetrode with the largest mean DS amplitude to select DS event timestamps. Across all tetrode recordings we detected 32,215 DS events in total (mean ± SEM: 441.3 ± 29.2 per day, from 73 recording days in 12 mice); in silicon-probe recordings we detected 15,067 DS events in total (mean ± SEM: 1676.1. ± 316.5 per day, from 8 recording days in 3 mice).

#### Sharp-wave ripple detection

For the LFPs of each pyramidal CA1 channel, we subtracted the mean across all channels (common average reference), band-pass filtered for the ripple band (80–250 Hz; 4th order Butterworth filter) and their envelopes (instantaneous amplitudes) were computed by means of the Hilbert transform. The peaks (local maxima) of the ripple band envelope signals above a threshold (5 times the median envelope of that channel) were regarded as candidate events. The onset and offset of each event were determined as the time points at which the ripple envelope decayed below half of the detection threshold. Candidate events passing the following criteria were determined as SWR events: (*i*) ripple band power in the event channel was at least twice the ripple band power in the common average reference (to eliminate common high frequency noise); (*ii*) each event had at least four ripple cycles (to eliminate events that were too brief); (*iii*) ripple band power was at least twice the supra-ripple band defined as 200-500 Hz (to eliminate high frequency noise, not spectrally compact at the ripple band, such as spike leakage artefacts). For events passing these criteria, the local maximum of each envelope was taken as the peak of the SWR, and these timestamps were recorded. On recording days with several tetrodes in the CA1 pyramidal layer, we used the tetrode with the largest mean ripple envelope amplitude to select SWR events. In tetrode recordings we detected 65,370 SWR events (mean ± SEM: 895.0 ± 82.3 per day, from 73 recording days in 12 mice).

#### Place maps

To generate place maps, we divided the horizontal plane of the recording enclosure into spatial bins of 1.4 × 1.4 cm to generate the spike count map (number of spikes fired in each bin) for each neuron and the occupancy map (time spent by the animal in each spatial bin) in each task session. All maps were then smoothed by convolution with a two-dimensional Gaussian kernel (s.d. = 1.2 bin widths). Finally, spatial rate maps were generated by normalizing the smoothed spike count maps by the smoothed occupancy map.

#### Spatial Information

The amount of spatial information conveyed by the spike train of a given cell was calculated using the formula proposed by Skaggs W. E. et al.^49^:

$$\;Information\;per\;spike\;=\sum_{i=1}^{N}p_{i}\frac{\lambda_{i}}{\lambda }\text{log}\;2\frac{\lambda_{i}}{\lambda }$$

where *i* = 1, 2… *N* represents each spatial bin of the environment, *p*_*i*_ is the probability of occupancy of bin *i, λ*_*i*_ is the mean firing rate in bin *i*, and *λ* is the mean firing rate of the cell over all spatial bins.

#### Peri-event time histograms (PETHs)

For analysis, we excluded all DS and SWR events that occurred within 50 ms of one another. We constructed PETHs over 400 ms windows, 200 ms either side of the peak DS amplitude or the peak of the SWR envelope, using a 1 ms bin width. The mean firing rate of each neuron was calculated during each 1 ms bin over the 400 ms window for each event. Z-scored firing rates were generated (over the DS-triggered or SWR-triggered average) separately for each neuron by calculating the mean and standard deviation over the 400 ms PETH:

$$z_{i}=\frac{\left(x_{i}-\bar{x}\right)}{S}$$

where z_i_ is the z-score at time bin *i, x*_*i*_ is the firing rate in time bin *i*, $\bar{x}$ is the mean firing rate across all time bins, and s is the standard deviation of the firing rate across all time bins. The z-scored firing rate of each neuron was then smoothed using a 3-point moving average to eliminate spurious peaks in low firing rate neurons. For a cell to be classified as significantly activated during DS and/or SWR events, the firing rate within ± 20 ms of the event peak had to be > 3 standard deviations (s.d.) above baseline (calculated as the mean firing rate over the 400 ms window). We also calculated the proportion of activated cells as a function of activation threshold (2 < *z-score* < 4; Figures S2H, S3E).

#### Current source density analysis

Current sources and sinks were estimated from LFP recordings taken from single-shank 64-channel silicon-probes spanning the somato-dendritic axis of CA1 principal cells and reaching the inferior blade of the DG. LFP signals were first down-sampled to 1250 Hz. The current source density ^50^ unscaled signal at time *t* and electrode *n, CSD*[*t*]*n*, was estimated as:

$$CSD{\left[t\right]}_{n}=-\left(LFP{\left[t\right]}_{n-1}-2\times LFP{\left[t\right]}_{n}+LFP{\left[t\right]}_{n+1}\right)$$

where *LFP*[*t*]_*n*−1_, *LFP*[*t*]_*n*_ and *LFP*[*t*]_*n*+1_ are the LFP signals at time *t* recorded from neighboring electrodes (*n−1* and *n+1* are the channels immediately above and below *n*, respectively, with 20 μm spacing between electrodes). The silicon-probe recording site in the pyramidal layer was identified as the one with largest ripple-band power. We defined the location of radiatum and lacunosum moleculare layers according to the sharp-wave and theta laminar profiles, as previously described ^48^. We sorted dentate spike events into Type 1 (DS_1_) or Type 2 (DS_2_) in the following way. First, we calculated the CSD estimates for all DSs at the peak of each event and used PCA to find the first two Principal Components from the resulting CSD traces. These principal components had as many dimensions as the number of silicon-probe channels (64). We then used a 2-component Gaussian Mixture Model to classify the events based on their projection onto the first two principal components. This consistently resulted in two event classes having the strongest sinks in different areas of the molecular layer. In line with previous research ^22,24^, we classified the events with the strongest sink in the outermost part of the molecular layer as DS_1_, and events with their sink closer to the granular layer as DS_2_. Based on CSD classification, event proportions were DS_1_: 0.35; DS_2_: 0.65 (5274 DS_1_ versus 9793 DS_2_, based on 15,067 events from 8 recording days in 3 mice).

#### Linear discriminant analysis classifier

To distinguish between DS_1_ and DS_2_ events using only the LFP traces, we trained a linear discriminant analysis (LDA) classifier using silicon-probe recorded LFPs from the granule cell layer (https://doi.org/10.5281/zenodo.10034433). LFP signals were first down-sampled to 1250 Hz and low-pass filtered at 50 Hz. We extracted 400 ms epochs centered around the peak of each DS (-200 to +200 ms, with 0.8 ms bin width), providing 500 time-based features (dimensions), one for each time bin, for each LFP trace. We then performed PCA on all silicon-probe-recorded DS LFP traces (15,067) to extract the number of components explaining 90% of the variance. This resulted in 16 principal components, which were then used to train a LDA classifier. We generated 20 models by, each time, randomly selecting 75% of the dataset, which was labelled as DS_1_ or DS_2_ based on the CSD classification described above, and then testing the classifier on the remaining (unlabeled) 25% of data. The classifier success rate was: median (IQR) = 85.4 (85.3-85.6) %. We then used the model with the highest accuracy to classify DS_1_ and DS_2_ events from LFPs recorded from the granule cell layer in our tetrode-recorded data. From tetrode-recorded LFPs, the proportions of Type 1 and Type 2 DS events were: median (IQR) DS_1_ = 0.34 (0.25-0.38); DS_2_ = 0.66 (0.62-0.75), based on 10,337 DS_1_ versus 21,740 DS_2_ events in 73 recording days in 12 mice.

#### Population spiking vectors

We generated event-based hippocampal population vectors of instantaneous principal cell spiking for every DS and SWR event using 50 ms wide windows centered on the peak of the DS or the peak envelope of the CA1 ripple (±25 ms from the peak). In addition, we calculated the spiking activity of hippocampal principal cells in equivalent 50 ms (‘no event’) control epochs, that contained neither DS nor SWRs. Baseline periods were selected from the same sleep sessions and excluded all epochs ± 250 ms either side of any DS or SWR events. To calculate the proportion of coactive neurons in each time window, we calculated the number of simultaneously active hippocampal principal cells (i.e., cells firing at least one spike during the 50 ms window) by the total number of simultaneously recorded hippocampal principal cells. We then calculated the mean proportion of coactive cells for each recording session. For inclusion in these analyses, each recording session required a minimum of 100 of each type of event (DS_1_, DS_2_, SWR) and a minimum of 20 simultaneously recorded hippocampal principal cells.

#### Population-level sparsity

The sparsity *S* of a given population firing vector *x* was calculated using the Gini index ^33,51,52^ as:

$$S=\frac{{\text{∑}}_{i=1}^{N}(2i-N-1)x_{i}}{N{\text{∑}}_{i=1}^{N}\;x_{i}}$$

where *x* is the population vector containing, in ascending order, the spike counts discharged by each principal cell in a 50 ms time window (centered on the peak of the SWR, DS), *N* is the length of that vector (i.e. the number of simultaneously recorded principal cells), and *i* is the rank of spike counts in ascending order. Population vectors where the total number of spikes is more evenly distributed between neurons have a lower Gini index (lower sparsity) than population vectors where the total number of spikes is concentrated in a few neurons (higher sparsity).

#### Logistic regression classifier

We used a logistic regression classifier to distinguish between population vectors of hippocampal principal cell spiking activity during DS, SWR, or equivalent duration (50 ms) control vectors that were taken from 200–250 ms periods before or after the peak of either the DS or SWR events. For each recording session, we generated matrices of these population vectors (cells × epochs) for these four different event-types, and then binarized the spike counts (i.e., spike count > 0 = 1, else 0) to control for the influence of firing rate differences between neurons. For each recording day, we used the event with the lowest number of epochs to determine the training set size – for example, if there were 200 DS events, we used 75% (150 population vectors) as the DS training set, and randomly subsampled the SWR matrix for 150 SWR population vectors (with identical principal cells). This way, the training input to the classifier was balanced across event types. Similarly, the testing set consisted of the remaining (unlabeled) 25% of population vectors from the DS population vectors plus an equivalent number of SWR population vectors (e.g., 50 DS population vectors and 50 SWR population vectors, subsampled from the remaining SWR testing matrix). For each recording day, we ran three models: one to classify event epochs, one to classify pre-event epochs and one to classify post-event epochs. Model accuracy was measured as the proportion of correctly classified events (DS versus SWR, or pre-DS versus pre-SWR, respectively).

#### Peer-to-peer coactivity analysis

We constructed hippocampal population graphs that represent the coactivity relationships between all pairs of principal cell spike trains recorded during a given sleep or exploratory session. These coactivity graphs were computed using 50 ms time windows for DS and SWR events and theta cycles as time windows for active exploratory sessions. To further control for the shared influence of the general network activity on peer-to-peer coactivity, we used for any two neurons (*i, j*) the regression coefficient *β*_*ij*_ obtained by fitting the GLM (Figure 3A):

$$x_{j}\;\sim \;\beta_{ij}x_{i}+\alpha_{ij}P$$

where *x*_*j*_, *x*_*i*_ are the z-scored event-nested spike trains of individual neurons *j* (the target) and *i* (the predictor), and *P* is the summed activity of the other *N* − 2 neurons,

$$P=\sum_{n=0}^{N-\{i,j\}}x_{n}$$

with *α*_*ij*_ weighting the influence of the population contribution to the activity of target neuron *j*. The recorded neurons (and their coactivity associations) are therefore the nodes (and their edges) in the coactivity graph of each task session. We described each graph by its adjacency matrix, *A*, as the *N* × *N* square matrix containing the pairwise coactivity relations within the network, yielding a weighted graph with no self-connections:

$$A=\left(\begin{array}{ccc} \beta_{0,0} & \cdots & \beta_{0,N}\\ \vdots & \ddots & \vdots \\ \beta_{N,0} & \cdots & \beta_{N,N} \end{array}\right)$$

with *β*_*i*,*i*_ = 0 ∀*i in N*, and the symmetry in the weights of the network being ensured by setting $A=\frac{A+A^{T}}{2}$ to form an undirected graph.

#### Clustering coefficient

We computed the clustering coefficient *C*_*i*_ to characterize the network’s local coactivity structure by scoring the triadic firing relationships established by each neuron *i* with the other neurons in the population, using the formula proposed by Onnela et al. ^53–55^:

$$C_{i}=\frac{{\text{∑}}_{jq}\;{\left(\hat{\beta_{lJ}}\hat{\beta_{lq}}\hat{\beta_{Jq}}\right)}^{1/3}}{k_{i}\left(k_{i}-1\right)}$$

where *j* and *q* are neighbors of neuron *i*, all edge weights are normalized by the maximum edge weight in the network $\hat{\beta }=\beta /\text{max}(\beta )$, and *k*_*i*_ is the degree of neuron *i*, which in these weighted graphs with no self-connection is equal to the number of neurons minus one. Note that this formula accounts for negative edges, yielding a negative value when there is an odd number due to the negative edges in the triad; it is positive otherwise. This method to assess firing relationships in the neuronal population of the hippocampus as a signed network where both positive and negative edges (i.e., correlated and anti-correlated spike trains) coexist leverages from past studies investigating community organization in social networks, indicating that triads represent the smallest motif capturing “structural balance” in patterns of peer-to-peer relationships ^56^.

#### Single-neuron coactivity strength

We defined the single-neuron coactivity strength as the average pairwise coactivity relation of a given node in a weighted graph. As a reference, the strength in a weighted graph can be compared to the degree in a binary graph, which accounts for the number of the node’s neighbors. Here, the strength *S*_*i*_ of a node *i* is the average across all the weights *β*_*ij*_ of the edges projected from that node:

$$S_{i}=\frac{{\text{∑}}_{j=0\;}^{N}\;\beta_{ij}}{N}$$

where *N* is the number of neurons *j* that node *i* projects to.

#### Population vector similarity

Population vectors of hippocampal principal cell spiking activity were generated for baseline, SWR, and DS events as described above, yielding separate (cell × event number) matrices of spike counts for each event-type. To remove potential biases caused by unequal numbers of events, we used the event-type with the fewest epochs to determine the final matrix size. For example, if there were 200 DS events in a given recording session, we randomly subsampled the SWR and baseline matrices to extract 200 SWR and 200 baseline population vectors (with identical principal cells) for comparison. Next we binarized these matrices (spike count > 0 = 1, else 0). Then we assessed the self-similarity for each event matrix (cells × event number) by computing the Pearson correlation coefficient for every pair of population vectors from the same event-type, and then calculating the mean across all of these correlation coefficients. As an alternative, we also calculated the Jaccard similarity coefficient (*J*), which measures the size of the intersection (i.e. overlap in active units) between pairs of population vectors (A, B), divided by the size of the union:

$$J(A,B)=\frac{\left|A\cap \;B\right|}{\left|A\cup \;B\right|}$$

#### Population dimensionality

We estimated the dimensionality of the principal cell population firing structure during SWRs and DSs from activity matrices that were matched for neuron identity and the number of DS and SWR events. We applied Principal Component Analysis (PCA) to each activity matrix, using the number of simultaneously recorded principal cells as the maximum number of components. Each matrix required at least 20 principal cells for inclusion in the analysis. We then extracted the number of components explaining 90% of the variance in these population vectors and scaled this by the total number of neurons in each matrix (Figures 3L and S4M,P). We also show dimensionality for a range of explained variance values (Figure S4N-O). Note that the ratio of DG to CA cells in these matrices did not significantly affect the dimensionality estimate (Supplemental Table S1).

#### Theta-cycle detection

Theta cycles were detected as described in Lopes dos Santos *et al*. (2023). Briefly, we used masked Empirical Mode Decomposition ^57^; https://pypi.org/project/emd/) to separate CA1 LFPs into oscillatory components termed intrinsic mode functions (IMFs). We delineated individual theta cycles from their troughs and peaks, i.e. the local maxima and minima of the theta IMF. Theta cycles were defined as peak-trough-peak sequences with trough-peak and peak-trough intervals between 31-100 ms and peak-to-peak distances between 71-200 ms. Note that this method is designed to detect chains of theta cycles but to do so it identifies each cycle independently.

#### Reactivation of waking coactivity patterns

We leveraged our pairwise peer-to-peer coactivity measure (as described above; Figure 3A) to estimate DS and SWR reactivation. With this, we compared the tendency of principal cell pairs to co-fire in theta cycles during exploration (theta coactivity) with the tendency to co-fire in DS (or SWR) during the following post-exploration sleep/rest period (post-DS or post-SWR co-firing), controlling for their baseline co-firing in the pre-exploration sleep/rest period before (pre-DS or pre-SWR co-firing) and mouse identity, using a linear mixed model:

$$\text{Post}\;\sim \;\beta_{0}+\beta_{theta}+\beta_{pre}+{\text{υ}}_{mouseID}+e$$

where *β*_0_ is the intercept of the regression line, *β*_*theta*_ is the regression coefficient for the theta co-firing, *β*_*pre*_ is the regression coefficient for the pre-exploration offline co-firing (in DS or SWR events), *υ*_*mouseID*_ is the individual mouse identity, and *e* the error term. Likewise, we compared the tendency of principal cell pairs to co-fire in theta cycles during exploration (theta coactivity) with the tendency to co-fire in DS or SWR during the pre-exploration sleep/rest period (pre-DS or pre-SWR co-firing), controlling for their post-exploration co-firing in the sleep/rest period after (post-DS or post-SWR co-firing) and mouse identity, using the reverse linear mixed model:

$$\text{Pre}\;\sim \;\beta_{0}+\beta_{theta}+\beta_{post}+{\text{υ}}_{mouseID}+e$$

From these LMMs, we extracted the β coefficients predicting post-SWR or post-DS coactivity from theta coactivity (controlling for pre-SWR or pre-DS coactivity, respectively) and tested their significance in two ways. First, we performed control GLMs using the pre-DS (or pre-SWR) coactivity as the dependent variable and the theta coactivity and post-DS (or post-SWR) coactivity as the independent variables. In these models, pre-event, post-event and theta coactivity were entered as fixed-effects and mouse identity as random-effects, using the restricted maximum likelihood method (implemented using the MixedLM class, and fit() method with default parameters, from the statsmodels library (Seabold and Perktold, 2010) in Python3.10). Second, we constructed a random probability distribution of β weights for theta coactivity by shuffling the cell pair identity, thereby generating a null distribution (based on 1000 LMMs, each time randomly shuffling cell-pair identity).

#### Closed-loop optogenetic interventions

For DS-informed interventions, real time detection of DSs was achieved by first high pass filtering the DG LFP signals (5 Hz) using the on-board signal processing capabilities of the Intan RHD evaluation board (RHD2000, Intan Technologies, USA) and triggering a laser pulse if the LFP signal exceeded a voltage-threshold. Thresholds for DS-onset detection were set for each mouse during a sleep session at the start of each recording day so that DS events were consistently detected (~3 S.D. above mean signal amplitude). Threshold detection triggered a digital transistor-transistor logic (TTL) output pulse from the RHD interface to a Master 8 stimulation timing device (A.M.P.I., Jerusalem, Israel), which in turn sent a 100 ms duration square-wave pulse to activate the laser. In the ‘DS-synchronized’ condition, the laser was triggered with zero latency from DS-onset, whereas in the ‘DS-delay’ condition the laser was triggered 100 ms after DS detection (Figures 5, 6). The rates of false negatives (DS not triggering laser pulse) and false positives (laser pulse emitted for LFP trace not meeting DS criteria) were 1.8±0.6% and 4.7±0.4%, respectively. The laser delivered yellow-green light (561-nm) into the dentate gyrus, which in DG^Grm2^::ArchT mice activated the outward proton pump, Archaerhodopsin T to suppress spiking activity in DG granule cells. To investigate changes in firing rates in individual hippocampal principal cells during light-delivery, we constructed PETHs over 400 ms windows, 200 ms either side of DS-onset, using a 1 ms bin width and extracted the peak firing rate during DS-synchronized light-delivery versus DSs with no light delivery. In addition, we z-scored the binned spike trains and calculated the mean z-score between DS-onset and 100 ms after DS-onset for each hippocampal principal cell during DS-synchronized light-delivery versus the equivalent 100 ms no-light period in the DS-delay condition.

For SWR-informed interventions, the Intan evaluation board was configured with firmware enabling additional filtering. Five operations were performed on the continuously acquired CA1 wideband LFP signal to provide a real time estimate of the instantaneous power in the ripple-band ^58,59^. (1) To enable low-latency processing, the signal was first down-sampled to 2.5 kHz by averaging the raw 20 kHz data stream with a sliding window of 8 samples with no overlap. (2) This signal was then high-pass filtered (using a 1st order digital infinite impulse response filter with a corner frequency of 1.6 Hz to remove amplifier offset and electrode drift). Next, the signal was (3) band-limited to 100–200 Hz with a 4th order Butterworth filter, (4) rectified by taking its absolute value, and (5) amplified 128-fold and smoothed with an exponential moving average operation over an equivalent window size of 32 samples (12.8 ms). To detect SWR events in this band-power estimate, the threshold level for each mouse was set during a sleep session at the start of each recording day to ensure consistent (~3 S.D. above mean power) detection throughout the day. On detecting a threshold crossing, the Intan recording controller delivered a 5 ms TTL pulse to a Master 8 stimulation timing device (A.M.P.I., Jerusalem, Israel). Analogous to the DS-informed interventions, in the ‘SWR-synchronized’ condition the laser was triggered with zero latency from SWR-onset, whereas in the ‘SWR-delay’ condition the laser was triggered 100 ms after SWR detection.

#### Recognition memory tasks

On each day of both the novel-object and novel-position recognition tasks, mice explored a square-walled open field (Figure 6A; the ‘object arena’) containing four objects, each positioned midway along a given wall, ~1cm from the wall edge. Objects used were ~3 × 3 × 4 cm (width × depth × height) objects (e.g., Lego™ blocks or other similar items). During the first session in the object arena, mice explored four completely novel objects (‘sampling’ session, 10 min). After the sampling session, mice were placed into a sleep box where they received DG-targeting light delivery that was either synchronized to event detection (DS-synchronized or SWR-synchronized condition) or delayed by 100 ms from event detection (DS-delay or SWR-delay condition), as described above (sleep/rest session, 20 min). In the novel object recognition task, before the start of the next test session, one of the four objects was replaced with a different (and completely novel) object, and mice then explored the four objects again (‘test 1’ session, 10 min). This process was repeated, with another sleep session (~20 min, with either DS-sync or DS-delay light-delivery), followed by another object exploration session with one completely novel object and three previously encountered objects (‘test 2’ session, 10 min). In the novel position recognition task, the locations of two of the initially sampled objects were swapped (e.g. North and West), whereas the other two objects remained in their original positions. In the novel position task, only DS-the synchronized and DS-delay conditions and only the first test session were used. During each test session, we measured the time spent exploring each object and we calculated the percentage time spent investigating the novel object (or novel positioned objects) versus the mean percentage time spent investigating the familiar objects (i.e., those objects seen in the previous session and/or those in the same locations). For analysis, four ‘object-zones’ were created by dividing the arena into nine equal sized square zones (~12 × 12 cm), such that four of these zones contained the objects. Time spent in the object zone was determined directly from the automated tracking data. Between sessions, the floor of the maze and the objects were cleaned with water. On any given day, mice received the same light-delivery condition.

#### Tone fear conditioning task

Fear conditioning was conducted in one of four operant chambers each with distinct visual cues (ENV-307A, Med Associates Inc., IN, USA). Mice were exposed to five auditory cues (either 2900 Hz tone or white noise, 72 dB, 30s duration), each co-terminating with a mild foot-shock (0.3 mA, 0.5 s). The mean ITI was 74s (range: 60 to 90s). Immediately after fear conditioning, mice were removed from the operant chamber and placed into the sleep box where they received DS-Sync or DS-Delayed DG cell silencing for 45 minutes. For the recall session, mice were then placed into a different operant chamber than the one where they received conditioning (to reduce the impact of contextual cues on recall). Mice were exposed to the same five auditory cues but no foot-shocks were given. Fear memory was assessed by measuring freezing responses during the first two cues presented in the recall session (before extinction occurs) and comparing these responses to freezing responses to the first cue during training (before any shocks were given). Freezing was measured using automated movement detection software (ezTrack, ^60^) and expressed as a % of tone duration (i.e. freezing for 15s during a 30s tone = 50% freezing).

#### Tissue processing and immunohistochemistry

At the completion of experiments, mice were deeply anesthetized with pentobarbital and perfused transcardially with 0.1 M PBS followed by 4% paraformaldehyde (PFA) in PBS. Brains were extracted and kept in 4% PFA for ~24–72 h and then transferred to PBS (with 0.05% sodium-azide). For tetrode localization, free-floating sections (50 μm) sections were mounted on slides and imaged at ×5 using a Zeiss microscope (AxioImager M2; Zeiss, Plan-Neofluar 5× /0.16 objective). For immunostaining, free-floating sections (50 μm) were rinsed in PBS with 0.25% Triton X-100 (PBS-T) and were blocked for 1 hour at ~20 °C in PBS-T with 10% normal donkey serum (NDS). Sections were then incubated with primary antibodies diluted in 3% NDS blocking solution and incubated at 4 °C for 72 hours (GFP anti-chicken, 1:1,000, Aves Labs, catalog no. GFP-1020; NeuN guinea pig, 1:500, Synaptic Systems, catalog no. 266 004). All sections were rinsed three times for 15 min in PBS-T and incubated for 4 hours at ~20 °C in secondary antibodies in the blocking solution (Cy3 donkey anti-guinea pig, 1:400, Jackson ImmunoResearch, catalog no. 706-165-148; goat anti-chicken 488, 1:1,000, Thermo Fisher Scientific, catalog no. A-11039). Sections were then rinsed three times for 15 min in PBS-T, with some sections then incubated for 1 min with DAPI (0.5 μg ml^−1^, Sigma, D8417) diluted in PBS to label cell nuclei before three additional rinse steps of 10 min each in PBS. Sections were mounted on slides, cover-slipped with Vectashield (Vector Laboratories, catalog no. H-1000) and stored at 4 °C. Sections were also used for anatomical verification of the tetrode tracks. Images were acquired using a Zeiss confocal microscope (LSM 880 Indimo, Axio Imager 2) with a Plan-Apochromat ×20/0.8 M27 objective and the ZEN (Zeiss Black 2.3) software.

### Quantification and Statistical Analysis

Analyses were performed in Python 3.8 (https://www.python.org/downloads/release/python-3816/) and Python 3.10 (https://www.python.org/downloads/release/python-31011/), using the Python packages DABEST (Ho *et al*., 2019), scipy (Virtanen *et al*., 2020), numpy (Harris *et al*., 2020), matplotlib (Hunter, 2007), seaborn (Waskom, 2021), pandas (McKinney, 2010), scikit-learn (Pedregosa *et al*., 2011), statsmodels (Seabold and Perktold, 2010). Error bars, mean ± S.E.M unless otherwise stated. We used throughout this study a bootstrap-coupled estimation of effect sizes, plotting the data against a mean difference between the left-most condition and one or more conditions on the right and compare this difference against zero using 5,000 bootstrapped resamples. In these estimation graphics (DABEST plots), each black dot indicates a mean difference and the associated black ticks depict error bars representing 95% confidence intervals; the shaded area represents the bootstrapped sampling-error distribution. Bandwidth estimates for the kernel density estimate were computed using the scikit-learn package. We used the DABEST package to calculate test statistics and p-values and visualize data. The test statistic is the mean difference and the p-value is the is the probability of observing the effect size (or greater), assuming the null hypothesis of zero difference is true. Paired permutation tests (or equivalent paired tests) were performed for repeated-measures analyses and unpaired tests used for independent samples. Data distributions were assumed to be normal, but this was not formally tested. Our results were replicable across mice and recording days. For the optogenetic interventions, the different closed-loop conditions (DS-Sync, SWR-Sync, DS-Delay, and SWR-Delay) were experienced in a randomized order across days. In the object recognition tasks, objects and their positions and the order of their replacement were randomized. Neural and behavioral data analyses were conducted in an identical way regardless of the identity of the experimental condition from which the data were collected, with the investigator blind to group allocation during analyses. No statistical methods were used to pre-determine sample sizes, but our sample sizes are similar to or larger than those reported in previous publications. Inclusion criteria for well-isolated single units were used as published in previous studies and are described in the corresponding subsections of the Method details. For the population vector analyses (Figures 3 and S4), each recording session required a minimum of 100 of each type of event (DS_1_, DS_2_, SWR) and a minimum of 20 simultaneously recorded hippocampal principal cells for inclusion.

## Key Resources Table

**Table:** 

REAGENT or RESOURCE	SOURCE	IDENTIFIER
**Bacterial and Virus Strains**		
AAV9-CAG-Flex-ArchT-GFP	UNC Vector Core	n/a
**Experimental Models: Organisms/Strains**		
C57BL/6J mice	Charles River	632
Grm2-Cre Tg(Grm2-cre)MR90Gsat/Mmucd	MMRRC	MMRRC_034611-UCD
Nestin-Cre B6.Cg-Tg(Nes-Cre)1Kln/J	Jackson Laboratories	IMSR_JAX:003771
**Software and Algorithms**		
Intan RHD2000	Intan Technologies	RHD2164
Positrack	Kevin Allen	n/a
Empirical Mode Decomposition in Python	Quinn A.J. et al. ^57^	n/a
Kilosort via SpikeForest	Magland J.F. et al. ^61^; Pachitariu M. et al. ^62^	n/a
**Other**		
12um tungsten wires	California Fine Wire	M294520
Optic fibers	Doric lenses	MFC_200/230- 0.37_25mm_RM3_FLT
Head-stage amplifier	Intan Technologies	RHD2164
561nm diode-pumped solid-state laser	Laser 2000	CL561-100

## Supplementary Material

Supplemental Figures and Tables

## Data Availability

The electrophysiology dataset reported in this study is being used in on-going projects and can be accessed under a data transfer agreement. We welcome enquiries for sharing it, please contact david.dupret@bndu.ox.ac.uk. This paper does not report original code. Any additional information required to reanalyze the data reported in this work paper is available from the Lead Contact upon request.
