# Supplementary material for: Offline hippocampal reactivation during dentate spikes supports flexible memory
Source: Neuron. Author manuscript; Available in PMC 2024 Oct 16. (PMC7616703; doi:10.1016/j.neuron.2024.08.022)
Supplement: Supplemental Figures and Tables [file EMS198381-supplement-Supplemental_Figures_and_Tables.pdf]

Supplemental Figures and Tables

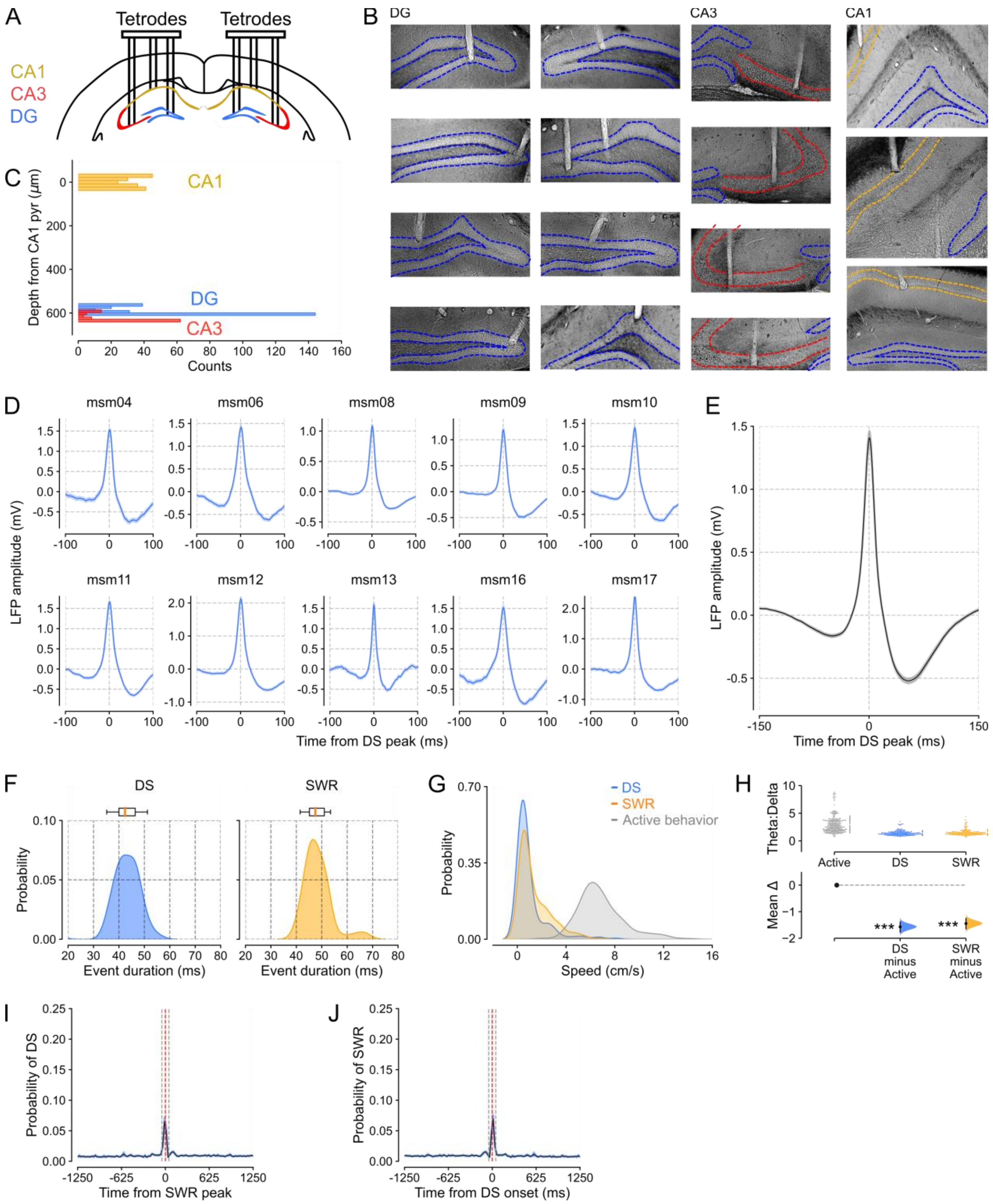

Figure S1. Tetrode locations and characterization of dentate spikes, related to Figure 1.
(A) Triple-(DG-CA3-CA1) tetrode layout schematic.
(B) Example histology showing tetrode tracks in DG, CA3, and CA1, with color-coded contours of the pyramidal cell layers and the granule cell layer.
(C) Histogram of tetrode locations in the dorsal-ventral plane (CA1 tetrodes n=176; DG tetrodes n=244, CA3 tetrodes n=92, from n=73 sessions, 12 mice).
(D) Examples of mean ± SEM dentate spike (DS) local field potential (LFP) waveforms from individual mice.

(E) Group mean ± SEM DS LFP waveform (n=73 sessions, 12 mice).
(F) Kernel density estimates (KDEs) and boxplots for DS (left) and SWR (right) event durations (based on 32,215 DS events and 65,370 SWR events, n=73 sessions, 12 mice).
(G) DS and SWR events occur when mice are asleep or in quiet rest and not when they are active. The graph shows KDEs for the probability of DS (blue) and SWR (orange) occurrence versus active behavior (gray) for a range of movement speeds. Active behavior was determined from the theta-to-delta ratio (>2.4) from the CA1 LFP during open field exploration.
(H) Estimation plot showing that DS and SWR events occur when the theta-to-delta ratio is low compared to active behavior. For this analysis, we defined a minimum activity level (movement speed > 3 cm/s) based on the active behavior movement speed distribution (panel F) to include 99% of the area under the curve. We then extracted the theta-to-delta ratio for speeds above this minimum and compared this distribution to the theta-to-delta ratio distributions during DSs and SWRs. Upper: raw data points (each point shows mean theta-to-delta power during one active behavior session or one sleep session), with the gapped lines on the right as mean (gap) ± s.d. (vertical ends) for each event. Lower: difference (Δ) in theta-to-delta ratio between active epochs versus DS and SWR epochs computed from 5,000 bootstrapped resamples and with the difference-axis origin (dashed line) aligned to active behavior (black dot, mean; black ticks, 95% confidence interval; filled curve, sampling-error distribution). The test statistic is the mean difference, shown on the y-axis of the lower plot. P-values are from unpaired permutation tests, active versus event, ***$P < 0.001$.
(I) Cross-correlogram of the probability of DS occurrence with respect to SWRs. Note that there was a small increase in the probability of DS around the time of a SWR, with more than 90% of DSs not occurring within ± 50 ms of a SWR. Red dashed lines show peak of event, gray dashed lines show ± 50 ms from peak.
(J) As for panel I but for SWR occurrence with respect to DSs.

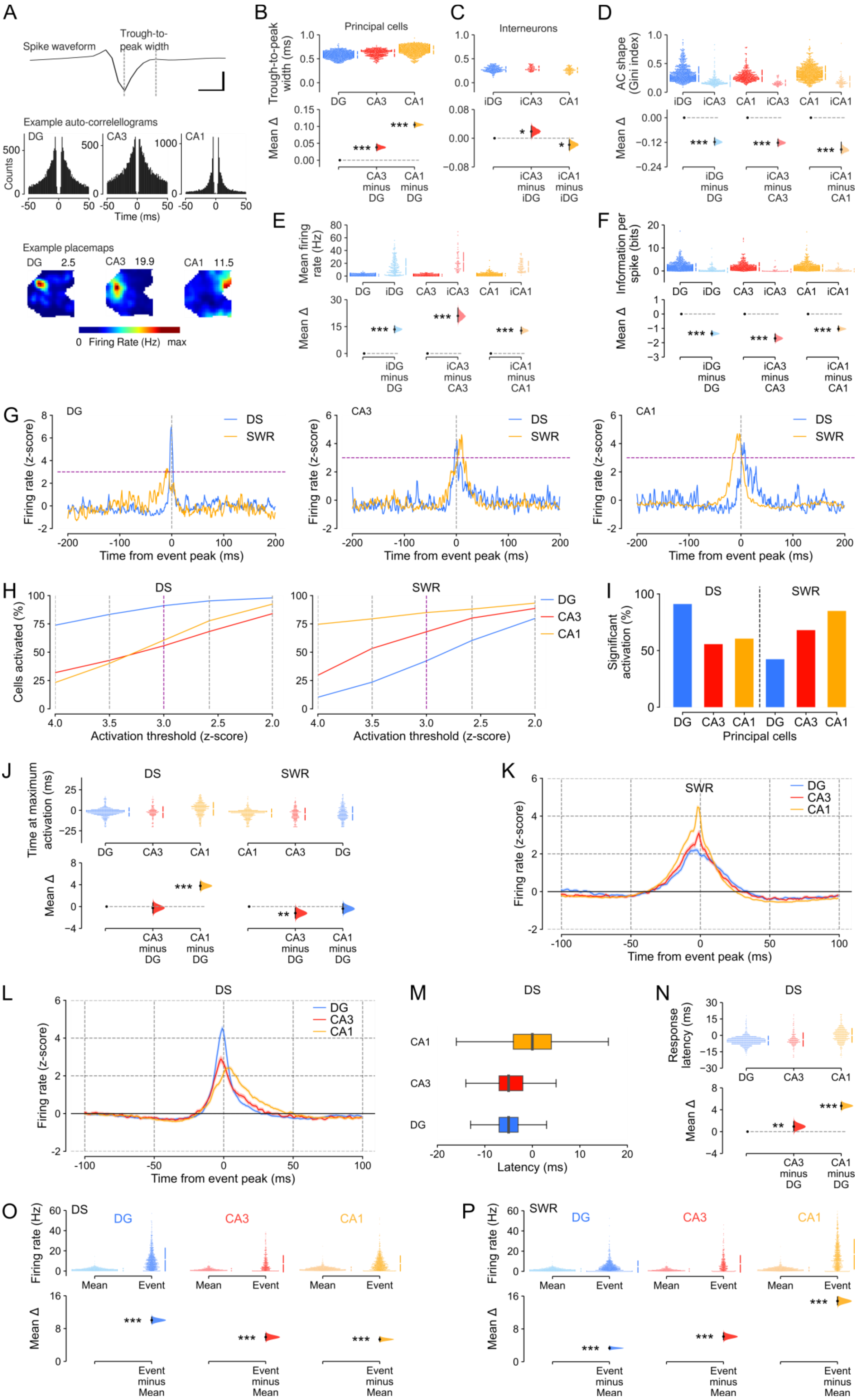
A
Spike waveform
Trough-to-peak width
Example auto-correlellograms
DG
CA3
CA1
Counts
Time (ms)
Example placemaps
DG 2.5
CA3 19.9
CA1 11.5
0 Firing Rate (Hz) max
B
Principal cells
Trough-to-peak width (ms)
DG
CA3
CA1
Mean Δ
CA3 minus DG
CA1 minus DG
C
Interneurons
iDG
iCA3
CA1
iCA3 minus iDG
iCA1 minus iDG
D
AC shape (Gini index)
iDG
iCA3
CA1
iCA3
CA1
iCA1
iDG minus DG
iCA3 minus CA3
iCA1 minus CA1
E
Mean firing rate (Hz)
F
Information per spike (bits)
G
Firing rate (z-score)
DS
SWR
Time from event peak (ms)
H
Cells activated (%)
Activation threshold (z-score)
I
Significant activation (%)
Principal cells
J
Time at maximum activation (ms)
K
L
M
Latency (ms)
N
Response latency (ms)
O
Firing rate (Hz)
Mean
Event
Event minus Mean
P

Figure S2. Dentate spikes activate hippocampal principal cells, related to Figure 1.
(A) Top: Example spike waveform showing the trough-to-peak measurement for spike width. Scale bar 100 µV and 0.5 ms. Middle: example auto-correlograms from individual DG, CA3 and CA1 principal cells. Bottom: example place maps from three individual (simultaneously recorded) DG, CA3 and CA1 principal cells illustrating the spatial distribution of spiking activity. The number in the top right corner shows the maximum firing rate of the cell in its place-field.
(B, C) Trough-to-peak width was used to classify principal cells versus interneurons. (B) Estimation plot showing that the trough-to-peak width is narrower in DG principal cells versus CA3 and CA1 principal cells. (C) Trough-to-peak width narrower in DG versus CA3 interneurons but is wider in DG versus CA1 interneurons. Note that the trough-to-peak width for DG principal cells remains wider for DG principal cells than DG interneurons.
(D) The auto-correlogram shape differs between principal cells and interneurons in DG, CA3 and CA1. Here, we used the Gini index to evaluate the sparsity in the spike probability distribution for each 1 ms bin of the auto-correlogram (between 0 and +50 ms). This distribution was more unequal for principal cells in all three regions, hence a higher Gini index; and was more equal for interneurons, hence a lower Gini index.
(E) The mean firing rate is higher in hippocampal interneurons (iDG, iCA3, iCA1: recorded cells with a trough-to-peak width < 0.4 ms) versus hippocampal principal cells.
(F) DG, CA3 and CA1 principal cells exhibit higher spatial information scores than interneurons in these respective regions.
For B-F estimation plots, Upper: raw data points (each point shows one principal cell); Lower: difference (Δ) in trough-to-peak width, firing rate, or spatial information (respectively) in DG versus CA3 and DG versus CA1. Other plot details as in Figure S1H.
(G) Examples of three individual principal cells' z-scored firing rates during DS (blue) and SWR (orange) events. The horizontal dashed line shows z-score = 3.
(H) Percentage of principal cells active during DS (left) versus SWR (right) events, as defined by crossing various z-thresholds.
(I) Percentage of significantly activated principal cells, as defined by a z-score > 3 (within ± 20 ms of the event peak, shown at time 0 in **G**, during DS (left) and SWR (right) events.
(J) Estimation plot showing the effect size for the differences in the time point of maximum neuronal spiking activity during DSs and SWRs in DG, CA3, and CA1 principal cells. Note that DG cells reached their peak firing significantly before CA1 neurons during DSs; CA3 cells reached their peak firing significantly before CA1 neurons during SWRs. Upper: raw data points (each point shows one principal cell that was significantly active (z > 3) during each event); Lower: difference (Δ) in time of peak activation in DG versus CA3 and DG versus CA1.
(K, L) Corresponding time course of principal cell instantaneous firing rate (z-score) during SWR (K) and DS (L) events.
(M, N) Using the time to cross the z-score > 3 threshold, we observed that during DS events, DG principal cells increase their firing activity significantly earlier than both CA3 and CA1 principal cells. Panel N is the corresponding estimation plot of the response latency (i.e. when each cell crossed the z > 3 threshold relative to the event peak) showing that DG cells are active before CA3 and CA1 principal cells during DSs. Upper and lower plots as in panel J.
(O, P) Estimation plots comparing the overall mean firing rate of each principal cell (calculated across the entire recording session) to its peri-event firing rate (calculated as the mean firing rate ± 5 ms around the peak of the event) during DS (O) and SWR (P) events. Upper: raw data points (each point shows one principal cell's mean rate and peri-event rate) in DG, CA3 and CA1; Lower: difference (Δ) in firing rate between mean rate and peri-event rate for DG, CA3 and CA1 separately. Other plot details as in Figure S1H.
For B-F,J,N-P, the test statistic is the mean difference, shown on the y-axis of each lower plot. P-values are from unpaired permutation tests, cell type versus cell type (B-F,J,N); or paired permutation tests, baseline (mean rate) versus event (O-P), **P* < 0.05, ***P* < 0.01, ****P* < 0.001.

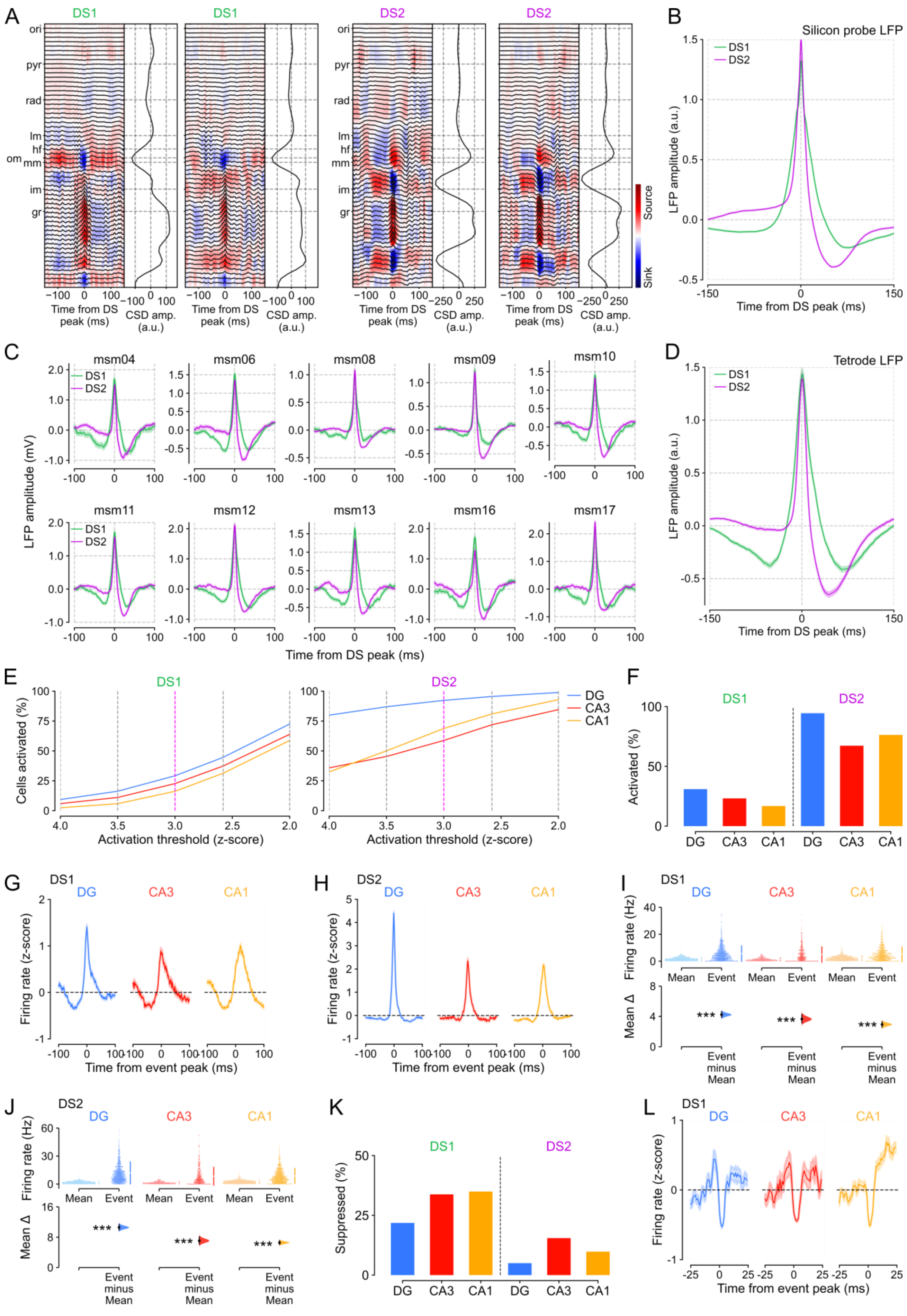

Figure S3. Current source density, local field potential profiles, and hippocampal principal cell spiking for DS_1 and DS_2 events, related to Figure 2.

(A) Examples of current source density (CSD) profiles for individual Type 1 (DS_1) and Type 2 (DS_2) dentate spikes (two DS_1 examples, two DS_2 examples) recorded from a 64-channel silicon-probe. *Left panel*: the instantaneous CSD ±150 ms around the event peak. *Right panel:* the CSD amplitude at each depth (based on the mean amplitude from –25 to + 25 ms around the event peak). Stratum oriens: ori; pyramidal layer: pyr; stratum radiatum: rad; lacunosum moleculare: lm; hippocampal fissure: hf; outer molecular layer: om; middle molecular layer: mm; inner molecular layer: im; granule-cell layer: gr.
(B) Group mean ± SEM LFP waveforms for DS_1 and DS_2 events from silicon-probe recordings (8 sessions, 3 mice).
(C) Examples of mean ± SEM LFP waveforms for DS_1 and DS_2 events from tetrode recordings in individual mice.
(D) Group mean ± SEM LFP waveforms for DS_1 and DS_2 events from tetrode recordings (73 sessions, 12 mice)
(E) Percentage of principal cells active during DS_1 (left) versus DS_2 (right) events, and as defined by crossing various z-thresholds.
(F) Percentage of significantly activated principal cells, as defined by a z-score > 3 (within ± 20 ms of the event peak), during DS_1 (left) and DS_2 (right) events.
(G-J) Peri-event time histograms (G,H) showing z-scored firing rates ± 100 ms around the event peak and estimation plots (I,J) comparing overall mean firing rate (calculated across the entire recording session) to peri-event firing rate (calculated as the mean firing rate ± 5 ms around the peak of the event) for all principal cells during DS_1 and DS_2 events. DG n=921, CA3 n=388, CA1 n=887 principal cells (12 mice). Upper and lower plots as described in Figure S2O-P.
(K) Percentage of suppressed principal cells (i.e., cells with a z-score < 0 during the event peak) during DS_1 and DS_2 events.
(L) Peri-event time histograms showing z-scored firing rates ± 25 ms around the event peak for the lowest quartile of activated principal cells (i.e., the 25% least activated / suppressed principal cells) during DS_1 events. (DG n=230, CA3 n=97, CA1 n=221 principal cells).
For I and J, the test statistic is the mean difference, shown on the y-axis of each lower plot. P-values are from paired permutation tests, baseline (mean rate) versus event, $^{***}P < 0.001$.

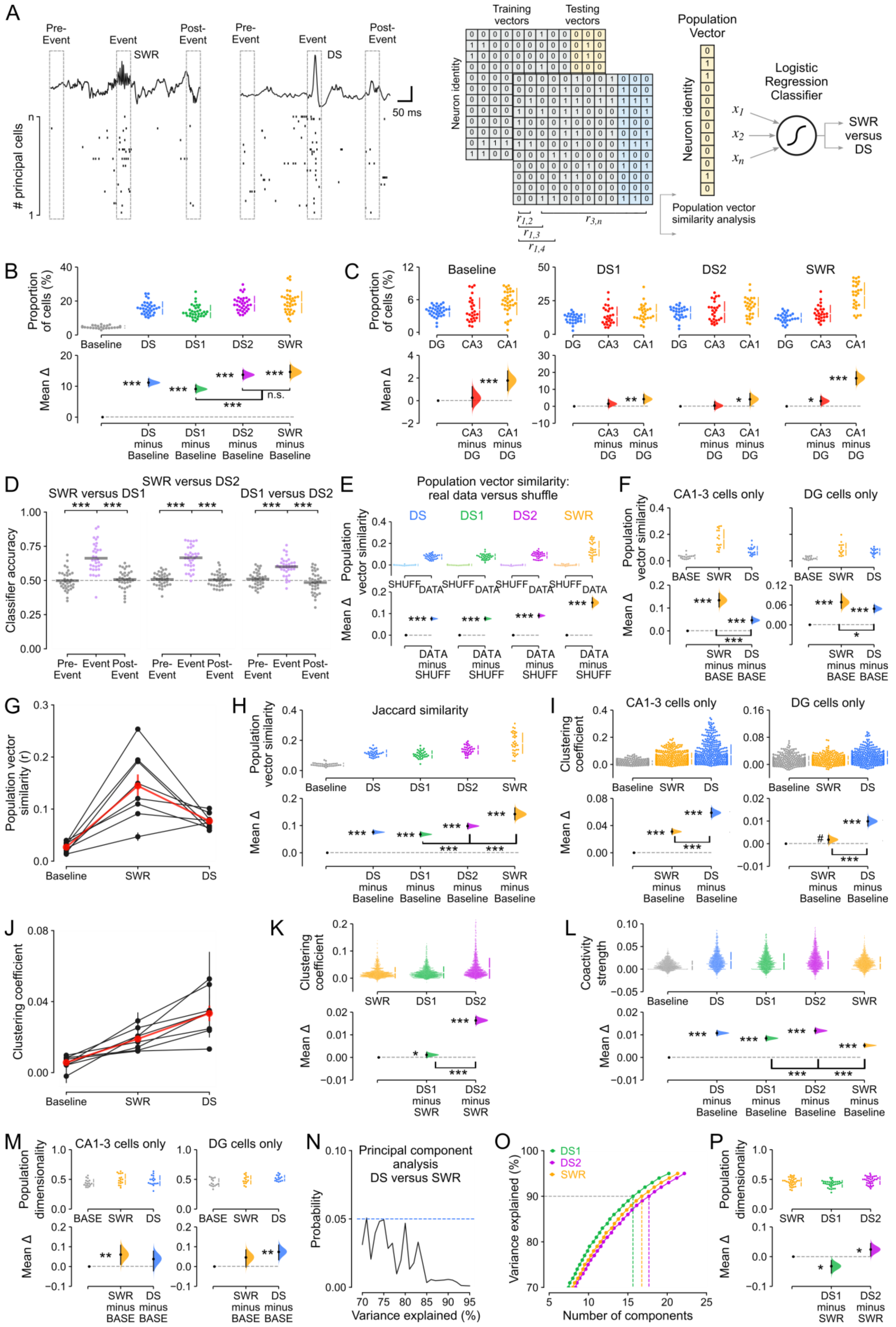
A
Pre-
Event
Event
SWR
Post-
Event
DS
50 ms
principal cells
n
1
Training
vectors
Testing
vectors
Neuron identity
Population
Vector
Logistic
Regression
Classifier
SWR
versus
DS
Population vector
similarity analysis
B
Proportion
of cells (%)
Baseline
DS
DS1
DS2
SWR
Mean Δ
n.s.
DS
minus
Baseline
DS1
minus
Baseline
DS2
minus
Baseline
SWR
minus
Baseline
C
Baseline
DS1
DS2
SWR
DG
CA3
CA1
CA3
minus
DG
CA1
minus
DG
D
SWR versus DS2
SWR versus DS1
DS1 versus DS2
Classifier accuracy
Pre-
Event
Event
Post-
Event
E
Population vector similarity:
real data versus shuffle
Population
vector similarity
SHUFF
DATA
DATA
minus
SHUFF
F
CA1-3 cells only
DG cells only
BASE
SWR
DS
SWR
minus
BASE
DS
minus
BASE
G
Population vector
similarity (r)
Baseline
SWR
DS
H
Jaccard similarity
I
CA1-3 cells only
DG cells only
Clustering
coefficient
J
Clustering coefficient
K
L
Coactivity
strength
DS1
minus
SWR
DS2
minus
SWR
M
CA1-3 cells only
DG cells only
Population
dimensionality
N
Principal component
analysis
DS versus SWR
Probability
Variance explained (%)
O
DS1
DS2
SWR
Variance explained (%)
Number of components
P
Population
dimensionality

Figure S4. Distinct population coactivity structures in DSs versus SWRs, related to Figure 3.
(A) Toy example illustrating construction of principal cell × event spiking activity matrices using the population firing vectors nested in 50 ms time windows centered on DSs, SWRs, or duration-matched (pre- and post-) control events. The proportion of active cells in each column vector (B-C) was defined as the proportion of cells with a non-zero spike count during the individual time window. Logistic regression classifiers (D) were trained using 75% of the population spiking activity vectors and tested with the remaining 25% of vectors, on any given session. We used the event type with the lowest number of epochs to determine the training and testing set size and then randomly subsampled the other event matrix to generate the same number of training and testing vectors for each event type, so that the classifier was balanced across event types. Separate matrices and classifiers were utilized for event, pre-event, and post-event epochs. For all of the analyses below, the inclusion criterion was that the session had to contain a minimum of 20 principal cells and 100 events of each type.
(B) Estimation plot showing the mean proportion of cells active during DS, DS_1, DS_2 and SWR events relative to equivalent duration-matched baseline periods during sleep when none of these events were present. Upper: raw data points (each point shows mean proportion of active cells in each vector across one recording session), with the gapped lines on the right as mean (gap) ± s.d. (vertical ends) for each event type. Lower: difference (Δ) in proportion of active cells between baseline epochs versus DS, DS_1, DS_2 and SWR epochs. Note that all events contained more active cells than baseline epochs but there was no statistical difference between the proportion of active cells in DS_2 versus SWR events.
(C) Likewise, shown is the proportion of cells active during Baseline, DS_1, DS_2 and SWR events split by hippocampal subregion. Upper: raw data points (each point shows mean proportion of active cells by subregion), with the gapped lines on the right as mean (gap) ± s.d. (vertical ends) for each subregion. Lower: difference (Δ) in proportion of active DG cells versus CA3 and CA1 cells.
(D) Classifier performance for SWR versus DS_1, SWR versus DS_2, and DS_1 versus DS_2 population vectors. Dashed line shows chance performance.
(E) Estimation plot showing the population vector similarity for all event types compared to their control condition in which each event vector was correlated with 'shuffled' population spiking vectors, where the cell identity was randomly shuffled. Upper: raw data points (each point shows mean population similarity for one recording session or shuffled equivalent), with the gapped lines on the right as mean (gap) ± s.d. (vertical ends) for each event. Lower: difference (Δ) in population similarity between real data for each event and its shuffled equivalent.
(F) Estimation plot showing population vector similarity for DS and SWR events compared to baseline control, as in Figure 3F, but separately for CA (left) and DG (right) principal cells. Upper: raw data points (each point shows mean population similarity by event type for one recording session), with the gapped lines on the right as mean (gap) ± s.d. (vertical ends) for each event type. Lower: difference (Δ) in population similarity between baseline epochs versus DS and SWR epochs.
(G) Our results showing significantly higher population vector similarity for DS and SWR events versus baseline, and for SWR versus DS events (Figure 3F) were recapitulated at the level of individual mice (8/8 mice for DS and SWR events versus baseline, 7/8 mice for SWR versus DS events; $p < 0.05$ permutation tests for DS versus baseline, SWR versus baseline, DS versus SWR with n=number of population vector pairs per mouse). Black traces show individual mice ± SEM (calculated on the number of population vectors per mouse); red trace shows the group mean ± SEM (calculated on n=8 mice).
(H) Our results using the Pearson correlation to compare population vector similarity (Figure 3F-G) were replicated using the Jaccard similarity measure. Estimation plot showing the population vector similarity for all event types compared to baseline epochs. Upper: raw data points (each point shows mean population similarity by event type for one recording session), with the gapped lines on the right as mean (gap) ± s.d. (vertical ends) for each event type. Lower: difference (Δ) in population similarity between baseline epochs versus DS, DS_1, DS_2 and SWR epochs.
(I) Estimation plot showing mean clustering coefficients (as Figure 3K) but for CA1-3 cells only (left panel) or DG cells only (right panel), which also show higher clustering coefficients for DS events.
(J) Our results showing significantly higher clustering coefficient for DS and SWR events versus baseline and DS versus SWR events (Figure 3K) were recapitulated at the level of individual mice (8/8 mice for DS and SWR events versus baseline, 7/8 mice for SWR versus DS events; $p < 0.05$ permutation tests for DS versus baseline, SWR versus baseline, DS versus SWR with n=number of neurons per mouse). Black traces show individual mice ± SEM (calculated on the number of neurons per mouse); red trace shows the group mean ± SEM (calculated on n=8 mice).
(K) Estimation plot showing that the neuronal coactivity graphs nested in both DS_1 and DS_2 events contained significantly stronger triads of coactive nodes compared to SWR graphs, as indicated by higher mean clustering coefficients. This was notably the case for DS_2.
(L) Single-neuron coactivity strength. As an alternative method to the triadic clustering coefficient parameter, we also show in this estimation plot that SWR and DS events differ in neuronal coactivity strength and from baseline epochs. We defined the single-neuron coactivity strength as the average

pairwise coactivity relation of a given neuron with its population peers. For any two neurons (i, j), we obtained the regression coefficient β from a generalized linear model predicting the spike discharge of neuron j from the activity of neuron i while regressing out the activity of the remaining population. The strength of neuron i is then the average across all the weights β_{ij}.

(M) Estimation plot showing population dimensionality required to explain 90% of the variance in DS and SWR events compared to baseline control separately for population vectors containing only CA (left) or DG (right) principal cells. Upper: raw data points (each point shows mean population dimensionality by event type for one recording session), with the gapped lines on the right as mean (gap) ± s.d. (vertical ends) for each event type. Lower: difference (Δ) in population dimensionality between baseline epochs versus DS and SWR epochs. Importantly, considering here DG and CA regions separately for the dimensionality analysis bears the caveat that, to comply with our criterion of at least 20 simultaneously recorded principal cells for each recording session, these analyses use far fewer recording days and the resulting neuronal population vectors are much smaller (n=17 days, n=25.8±1.0 cells per vector for CA; n=15 days, n=25.5±0.9 cells per vector for DG) than the data presented in Figure 3K (n=34 days, n=37.2±1.8 cells per vector), which limits the comparison.

(N) PCA to compare the dimensionality of SWR versus DS matrices (cell × event number), matching the number of events for each event type, determining the number of components required to explain 70–95% of the variance. In each case, the dimensionality was significantly higher for DS versus SWR events at $\alpha < 0.05$ (Wilcoxon test for paired samples, one-tailed).

(O-P) The number of components required to explain equivalent amounts of variance was lower in DS_1 versus SWR and DS_2, and higher in DS_2 versus SWR events (N); as illustrated by the estimation plot in panel O, showing that a lower number of principal components was required to explain 90% of the variance across the population vectors nested in DS_1, compared to SWR and DS_2 events.

For B-F,H-I,K-M,P, the test statistic is the mean difference, shown on the y-axis of each lower plot. P-values are from paired permutation tests, baseline versus event (B,F,H,I,L,M), event versus pre-event and event versus post-event (D), data versus shuffle (E), event versus event (K,P); or unpaired permutation tests, cell type versus cell type (C), *$P < 0.05$, **$P < 0.01$, ***$P < 0.001$.

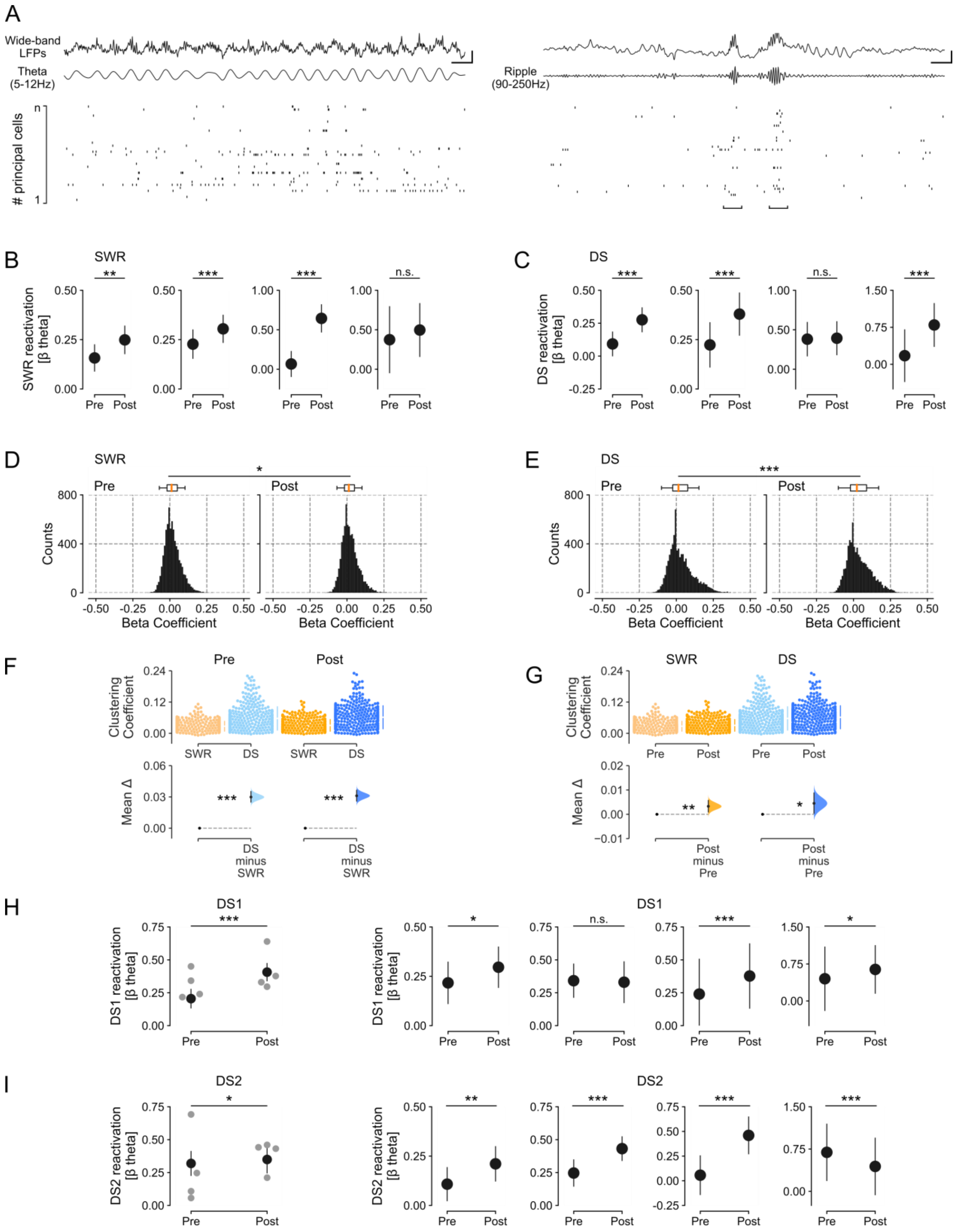

Figure S5. Offline reactivation of theta population coactivity in individual mice, related to Figure 4. (A) Raw data examples of hippocampal principal cells spiking activity during active exploration marked by theta oscillations (Left) and offline sleep/rest (Right; example SWRs shown). Scale bars 0.5 mV and 150 ms (Left) or 50 ms (Right). We applied our peer-to-peer coactivity analysis (Figure 3A) to obtain the waking patterns of population coactivity nested in theta cycles during exploration, and the offline patterns of population coactivity nested in either DS or SWR events during sleep/rest before and after exploration. With these, we next computed DS and SWR reactivation by measuring the tendency of the peer-to-peer theta firing associations to reoccur in post-exploration sleep/rest DS (or SWR) events, while controlling for prior pre-exploration DS (or SWR) coactivity, using a linear mixed model (Figure 4A).

(B) SWR reactivation in individual mice (measured by the β coefficients of the linear regression that predicted post-exploration SWR coactivity from waking theta coactivity, controlling for pre-exploration SWR coactivity).
(C) DS reactivation in individual mice (measured by the β coefficients of the linear regression that predicted post-exploration DS coactivity from waking theta coactivity, controlling for pre-exploration DS coactivity).
(D-E) Distribution of peer-to-peer coactivity values (β coefficients) for SWRs (D) and DSs (E) in the pre- and post-exploration sleep/rest sessions. Significance was tested using the Wilcoxon test for matched pairs: post > pre for DSs (U=1198580, n=7310, $p < 0.001$); and for SWRs (U=12839635, n=7310, $p < 0.001$).
(F-G) Estimation plots showing that the neuronal coactivity graphs nested in DSs contained significantly stronger triads of coactive nodes compared to SWR graphs, as indicated by higher mean clustering coefficients, during both pre- and post-exploration sleep sessions (F). Also, DSs and SWRs contained significantly stronger triads of coactive nodes during post- versus pre-exploration sleep sessions (G).
(H) Mean DS_1 reactivation pooled across mice (left panel) and in individual mice (right four panels; measured by the β coefficients of the linear regression that predicted post-exploration DS_1 coactivity from waking theta coactivity, controlling for pre-exploration DS_1 coactivity)
(I) As H but for DS_2 reactivation.
For F and G, the test statistic is the mean difference, shown on the y-axis of each lower plot. P-values are from paired permutation tests, event versus event (F), pre versus post (G), $^*P < 0.05$, $^{**}P < 0.01$, $^{***}P < 0.001$.

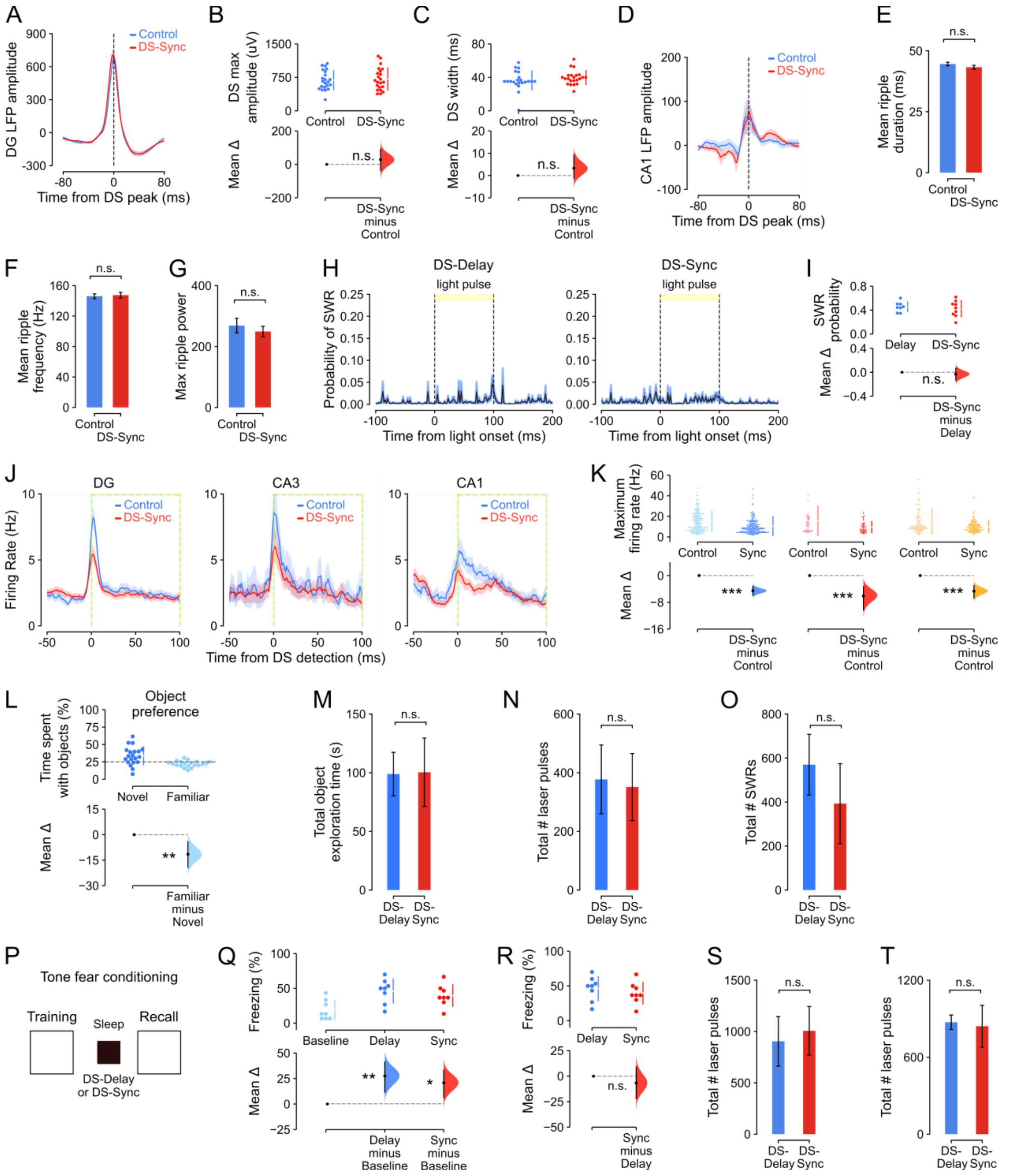

Figure S6. Closed-loop optogenetic suppression of dentate granule cells during DSs, related to Figures 5 and 6.

(A-C) During DS-triggered DG light-delivery for the optogenetic silencing of DG cells, there was no difference in the amplitude (A, B) or duration (C) of DSs in light-on versus light-off (control) DS events.
(D-I) DG light-delivery did not affect CA1 LFPs (D), ripple duration (E), intra-ripple frequency (F), ripple power (G), nor the probability of ripple occurrence (H-I; 22 recording sessions in 8 mice).
(J) DS-triggered DG light-delivery significantly reduced firing rates in DG, CA3 and CA1 principal cells relative to DSs with no light delivery (Control), in a paired analysis (same cells under both conditions). Each panel shows mean ± SEM firing rate. Average firing rates across the entire recording session (including sleep and exploration epochs) were: DG: 1.4±0.1 Hz, CA3: 1.7±0.3 Hz, CA1: 1.5±0.1 Hz.
(K) Estimation plot showing the maximum firing rate (during DS events) in DG, CA3 and CA1 principal cells during DS-Sync or Control (DSs with no light delivery), as in panel J. Upper: raw data points (each

point shows maximum firing rate), with the gapped lines on the right as mean (gap) ± s.d. (vertical ends) for each event type. Lower: difference (Δ) in maximum firing rates between DS-Sync and no-light control condition (paired permutation test in DG: n=216 cells, CA3: n=50 cells, and CA1: n=133 cells from n=22 recording sessions in 8 mice).

(L) Estimation plot for object preference in a 'no-laser' control group of mice, showing significantly more time spent investigating the novel object (n=20 test sessions in 5 mice).

(M-O) In the continuous novel object recognition task, the total time spent exploring the objects (M), the number of laser pulses delivered (N) and the number of SWRs detected during sleep sessions (O) did not differ between the DS-delay and DS-sync conditions.

(P-S) The tone fear task in which mice had 5 tone-shock pairings during conditioning followed by either DS-Delayed or DS-Sync stimulation, and then fear memory recall. Mice froze more during recall than the baseline (Q), but this did not differ between the DS-Delay and DS-Sync groups (R; n=8 sessions in 4 mice). Mice received an equivalent number of laser pulses in the two groups (S).

(**T)** In the novel position recognition task, mice in the DS-Delayed and DS-Sync groups received equivalent numbers of laser pulses.

E-G, M-O, S,T show mean ± SEM. For B,C,I,K,L,Q,R, the test statistic is the mean difference, shown on the y-axis of each lower plot. P-values are from paired permutation tests, Control (no laser) versus DS-Sync (B,C,I,K,R), Novel versus Familiar (L), Baseline versus DS-Delay and DS-Sync (Q), *$P < 0.05$, **$P < 0.01$, ***$P < 0.001$.

Table S1. Ratio of DG to CA neurons influence on the dimensionality and similarity of population firing vectors, related to Figure 3.

Event	Dependent variable	Independent variables	Degrees of freedom	r-value	p-value
DS	Dimensionality	Ratio of DG:CA neurons	33	0.24	0.18
DS1	Dimensionality	Ratio of DG:CA neurons	33	0.12	0.51
DS2	Dimensionality	Ratio of DG:CA neurons	33	0.26	0.14
SWR	Dimensionality	Ratio of DG:CA neurons	33	0.32	0.07
DS	Similarity	Ratio of DG:CA neurons	33	-0.06	0.75
DS1	Similarity	Ratio of DG:CA neurons	33	-0.13	0.46
DS2	Similarity	Ratio of DG:CA neurons	33	-0.08	0.66
SWR	Similarity	Ratio of DG:CA neurons	33	-0.39	0.02

Table S2. Linear mixed model analysis for SWR and DS reactivation of waking theta coactivity patterns, related to Figure 4.

Event	Dependent variable	Independent variables	No. Observation
SWR	Post-exploration	Theta coactivity<br>Pre-exploration	7310
SWR	Pre-exploration	Theta coactivity<br>Post-exploration	7310
DS	Post-exploration	Theta coactivity<br>Pre-exploration	7310
DS	Pre-exploration	Theta coactivity<br>Post-exploration	7310

Event	Dependent variable	Independent variables	β coefficient	CI (95%)	z	Prob.
SWR	Post-exploration	Theta coactivity	0.37	[0.32, 0.43]	13.1	P < 0.0001
		Pre-exploration	0.11	[0.09, 0.13]	9.5	P < 0.0001
SWR	Pre-exploration	Theta coactivity	0.20	[0.15, 0.26]	7.0	P < 0.0001
		Post-exploration	0.11	[0.04, 0.08]	9.5	P < 0.0001
DS	Post-exploration	Theta coactivity	0.39	[0.31, 0.46]	9.7	P < 0.0001
		Pre-exploration	0.25	[0.23, 0.27]	23.3	P < 0.0001
DS	Pre-exploration	Theta coactivity	0.24	[0.16, 0.32]	5.8	P < 0.0001
		Post-exploration	0.27	[0.25, 0.30]	23.3	P < 0.0001

Table S3. Counts of principal cells by session and mouse, related to Figures 1-6.

Figure number / panel	# sessions	# mice	# principal cells (mean ± SEM per mouse)		
			**DG**	**CA3**	**CA1**
Figure. 1F,G	73	12	921<br>(76.5±22.2 per mouse)	388<br>(32.3±9.6 per mouse)	887<br>(73.6±17.9 per mouse)
Figure. 2A-C	8	3	n/a	n/a	n/a
Figure. 2E-G	73	12	921<br>(76.5±22.2 per mouse)	388<br>(32.3±9.6 per mouse)	887<br>(73.6±17.9 per mouse)
Figure. 3B-D,F-G,K,L	34	8	647<br>(85.6±28.9 per mouse)	169<br>(25.6±9.1 per mouse)	449<br>(77.9±23.0 per mouse)
Figure. 4B-C	9	4	114<br>(28.5±7.2 per mouse)	5<br>(1.25±1.1 per mouse)	232<br>(58.0±18.0 per mouse)
Figure. 5F-G	43	9	548<br>(60.9±11.8 per mouse)	n/a	n/a
Figure. 5I-J	13	3	181<br>(60.3±14.0 per mouse)	n/a	n/a
Figure. 6H (DS)<br>Figure. 6H (SWR)	10<br>12	3<br>3	79 (26.3±10.3 per mouse)<br>173 (57.7±15.7 per mouse)	22 (7.3±3.8 per mouse)<br>32 (10.7±6.4 per mouse)	73 (24.3±4.0 per mouse)<br>68 (22.7±8.1 per mouse)
